# Conditional autoencoder asset pricing models for the Korean stock market

**DOI:** 10.1371/journal.pone.0281783

**Published:** 2023-07-31

**Authors:** Eunchong Kim, Taehee Cho, Bonha Koo, Hyoung-Goo Kang

**Affiliations:** 1 Business School, Hanyang University, Seoul, Republic of Korea; 2 Qraft Technologies, Seoul, Republic of Korea; 3 School of Business, Chungnam National University, Daejeon, Republic of Korea; Bucharest University of Economic Studies: Academia de Studii Economice din Bucuresti, ROMANIA

## Abstract

This study analyzes the explanatory power of the latent factor conditional asset pricing model for the Korean stock market using an autoencoder. The autoencoder is a type of neural network in machine learning that can extract latent factors. Specifically, we apply the conditional autoencoder (CA) model that estimates factor exposure as a flexible nonlinear function of covariates. Our main findings are as follows. The CA model showed excellent explanatory power not only in the entire sample but also in several subsamples in the Korean market. Also, because of this explanatory power, it can better explain market anomalies compared to the traditional asset pricing models. As a result of examining investment strategies using pricing error, the CA model measures the expected return of stocks better than the traditional asset pricing model. In addition, the CA model indicates that the firm characteristic variables are important in asset pricing conditional on macro-financial states, such as the global financial crisis and the coronavirus disease 2019 pandemic. The result shows that the major variables considered in the explanation of stock returns through the CA model may vary depending on the time. This is expected to provide a broader perspective on asset pricing through the CA model in the future.

## 1. Introduction

Various risk factors have been introduced in the extant literature to explain cross-sectional stock returns or market anomalies that traditional asset pricing models fail to elucidate [1–3]. These efforts have led to the inclusion of numerous factors, and this phenomenon of factor overflow is called factor zoo [4]. Indeed, scholars have endeavored to identify factors that provide information orthogonal to other existing factors, considering the multi-dimensionality of numerous factors. Thus, distinguishing between significant and unnecessary pricing factors becomes essential for identifying the true factors.

Alternatively, rather than specifying factors in advance based on the empirically observed cross-sectional characteristics, researchers have attempted to discover potential factors that can best explain the stock returns through ex-post and bottom-up approaches. A popular idea is to estimate latent factors by extracting information from high-dimensional data using machine learning. Such attempts to use latent factors for asset pricing began with Ross’ arbitrage pricing theory [5], which is extended in several studies using the principal components analysis (PCA) to analyze stock returns [6–8]. Enhancing PCA models by including only static observable factors, Kelly *et al*. [9] proposes an instrumented PCA (IPCA) asset pricing model, which includes unobservable latent factors that change over time. Inspired by the IPCA model, Gu *et al*. [10] proposed a conditional autoencoder (CA) asset pricing model to reflect the influence of external variables. The autoencoder, one of the machine learning methods, is a dimension reduction technique that can infer nonlinear relationships among data., that is, it is a generalized PCA with nonlinearity [11]. Gu *et al*. [10] find that the CA model exhibits a better explanatory power for stock returns than the traditional Fama-French factor (FF) models and IPCA models for the U.S. market. These results indicate that the latent factors estimated through machine learning techniques can be effectively employed in asset pricing models.

Using machine learning techniques, we examine the explanatory power of the CA model for the Korean market. To the best of our knowledge, this is the first study to apply the CA model to the Korean market and compare the explanatory power of the CA model with that of the traditional asset pricing models under different macro-financial.

The main results can be summarized as. First, the explanatory power of the CA model dominates that of traditional models. The out-of-sample (OOS) R^2^ for individual stocks and the test-asset portfolios constructed with each firm characteristic are larger for the CA model than for the traditional asset pricing model (e.g., FF models). Second, the OOS mispricing is much smaller in the CA than in the FF models. The number of statistically significant alphas in the CA model is smaller than that in the FF models. Therefore, the latent factors in the CA model demonstrate larger dispersion efficiency than the observable factors in the FF models in explaining heterogeneous stock returns. Third, the expected rate of return, calculated from the traditional asset pricing model, is compared with that based on the CA model in order to find out whether the CA model is more suitable in the Korean market or not. It shows that the benefits of the traditional asset pricing model are ambiguous for all strategies which utilize the price error. The result of overvalued (or undervalued) stocks is also not statistically significant. Our results indicate that the traditional asset pricing model is not valid in the Korean market. Finally, we identify the importance rankings of firm characteristics, which are not included in the existing asset pricing models. Furthermore, we find those firm characteristics that are important in asset pricing differ depending on specific periods, such as the global financial crisis or the COVID-19 pandemic. This finding is reported for the first time in the literature.

Our empirical analysis follows the study of Gu *et al*. [10], with several key differences. First, we use Korean market data to test the CA model. The test result confirms that the CA model, in general, performs well on stock data from countries other than the United States. Second, we compare the explanatory power of existing models in various subsamples. In a subsample in which the explanatory power of the existing asset pricing model is low, the CA model showed better explanatory power. Third, the investment strategies based on the pricing error of the model and calculated with the traditional asset pricing model or the CA model, are compared. The result reveals that the CA model’s profit is superior to that of the traditional asset pricing model. That would indicate that the CA model can be more accurate to identify undervalued stock (or overvalued stock). Therefore, the CA model can supplement the limitations of the existing traditional asset pricing models. This study confirms the superiority of the asset price model by the artificial intelligence model and indicates that it can be used in various financial fields.

The remainder of this paper is organized as follows. In Section 2, literature reviews. In Section 3, we describe the data and methodology. In Section 4, we present the empirical results, while in Section 5, we conclude the paper.

## 2. Literature review

Our paper also contributes to the growing field of machine learning in finance. Recent literature uses machine learning in finance including equity return forecasting, asset pricing, and risk management. For example, distinguished by algorithms, researchers tested other approaches with shrinkage methods [12, 13] the class of Support Vector Machines [13–16], as well as tree-based methods [17, 18] such as the Gradient Boosting Machine or the Random Forest. Furthermore, many papers applied various architectures of neural networks to predict future asset prices [19–21]. Other, less widespread methodologies include natural language processing [22] Principal component analysis [23], autoencoders [24], and Reinforcement learning [21, 25]. The use of artificial intelligence continues in the study of asset price models. Most applications, in line with the traditional asset pricing literature, consider only linear relationships between financial variables and subsequent stock returns. For example, the Capital Asset Pricing Model (CAPM) introduced by Sharpe [26], Lintner [27], and Mossin [28] posits that, in equilibrium, a stock’s expected return is solely driven by its sensitivity to a systematic risk factor, i.e., the market risk. An assumption is that the underlying pricing kernel is linear in only a single factor, i.e., the market portfolio.

Various studies, however, report violations of this assumption (e.g., Hou *et al*. [3], for a comprehensive list of asset pricing anomalies) and examine the alternative asset pricing models. Following Dittmar [29], we classify them into two subcategories. The first subcategory utilizes other pricing factors in addition to the market portfolio. Most prominently, Fama and French [30] propose a multifactor alternative to the CAPM and find that it is better at explaining cross-sectional variation in expected returns than the CAPM. Other examples include Ross’ asset pricing theory (APT) [5] and Merton’s intertemporal CAPM (ICAPM) [31]. The second subcategory abandons the restriction that the pricing kernel must be linear in pricing factors. Bansal *et al*. [32], Bansal and Viswanathan [33], Chapman [34], Dittmar [29], and Asgharian and Karlsson [35], among others, explore various nonlinear pricing kernels and show that such specifications outperform linear counterparts.

While the first subcategory of models motivates the use of multiple pricing factors, the second subcategory suggests that using interactions between these factors and incorporating nonlinear relationships between price-related variables and expected stock returns add incremental explanatory power. For this, many studies use machine learning methods. Messmer [36] and Feng *et al*. [37] predicted stock returns with neural networks. Bianchi *et al*. [38] used a machine learning method to predict bond yields and compared them with the existing traditional methods. Freyberger *et al*. [39] estimated the risk premium of stock returns through a non-linear additive function using the Lasso selection method. Feng *et al*. [37] impose a no-arbitrage limit using a predefined set of linear asset pricing factors and measure the loading of each of the above factors through a deep neural network. Rossi [40] derived a conditional mean-variance efficient portfolio based on the market portfolio and risk-free assets through Boosted Regression Trees. More recently, new methods have been developed to extract statistical asset pricing factors from large panels with various derivatives of principal component analysis (PCA). The Risk-Premium PCA, suggested by Lettau and Pelger [23], introduced a pricing error penalty to detect weak factors which explain the cross-sectional variance of returns. The high-frequency PCA, suggested by Pelger [41], utilized high frequency data to estimate local time varying latent factors.

Enhancing PCA models by including only static observable factors, Kelly *et al*. [9] proposes an instrumented PCA (IPCA) asset pricing model, which includes unobservable latent factors that change over time. The IPCA model can explain the stock return better than the PCA models or the FF models for the U.S. stock markets; IPCA renders the pricing errors (alphas) of many firm characteristic-managed portfolios insignificant. Inspired by the IPCA model, Gu *et al*. [10] proposed a conditional autoencoder (CA) asset pricing model to reflect the influence of external variables. The autoencoder, one of the machine learning methods, is a dimension reduction technique that can infer nonlinear relationships among data., that is, it is a generalized PCA with nonlinearity [11]. Gu *et al*. [10] find that the CA model exhibits a better explanatory power for stock returns than the FF and IPCA models for the U.S. market. These results indicate that the latent factors estimated through machine learning techniques can be effectively employed in asset pricing models.

The most recent study, Gu *et al*. [10], extended the linear conditional factor model of Kelly *et al*. [9] to a non-linear factor model using an autoencoder neural network. In this study, the model of Gu *et al*. [10] is replicated in the Korean market, and the explanatory power of the asset price model constructed using the machine learning method is checked whether it can be generalized.

## 3. Data and methodology

This chapter describes the scheme and the evaluation of the CA model. The data used for the CA model, the structure of the CA model, and the evaluation are elaborated. Comparative models are also described. S1 Fig in S1 Appendix is a schematic diagram of the CA model learning process. The methodology used in this study consists of four steps.

It is data collection in first step. Market price and fundamental data of KOSPI and KOSDAQ stocks are collected in the Korean market. The second step is the data preprocessing step for analysis. Merge each data and create a firm characteristics variable to use in analysis. It divides learning, verification, and test data necessary for AI model learning, and separates samples for subsample test. Step 3 is model training. With the training data generated in step 2, the CA model described in Section 3.2 is trained. The final model is created through hyperparameter tuning through the validation data. Step 4 is evaluation. In this step, the main findings of the study are drawn. Analyze model explanatory power, portfolio alpha test, APT strategy performance comparison, and importance of company-specific variables. In this chapter, the main parts of this process are explained in detail.

### 3.1. Data

We analyze the monthly data of common stocks listed on the KOSPI and KOSDAQ markets from January 1991 to December 2020. The monthly stock return is calculated using the adjusted stock price, reflecting dividends and par split. To eliminate survival bias, we include all firms that have been closed. Our sample includes 512,883 firm-months, with 1,425 firms per month on average. Additionally, following Gu *et al*. [10], we do not exclude financial firms or low-priced stocks. For each firm, we have 38 firm characteristic variables. Although Gu *et al*. [10] use 94 firm characteristic variables, we can only access quarterly data after 2000 for the Korean market because of data limitations and accounting differences. In comparison, Kelly *et al*. [9] employs 36 firm characteristic variables in their IPCA model.

Table 1 describes the 38 firm characteristic variables used in our empirical analysis. The first category (1–14) comprises monthly variables: market beta (beta) [40], market beta squared (betasq) [42], change in 6-month momentum (chmom) [43], the ratio of the current price to the 52-week high price (high52) [44], idiosyncratic return volatility (idiovol) [45], illiquidity (ill) [46], maximum daily return (maxret) [47], 1-month momentum (mom1m) [48], 6-month momentum (mom6m) [48], 12-month momentum (mom12m) [49], 36-month momentum (mom36m) [48], market equity (mvel1) [50], return volatility (retvol) [51], and total skewness (ts) [52].

**Table 1:** Definition of the firm characteristic variable. This table reports firm characteristics and their definitions. The first (1–14) and second categories (15–38) comprise monthly variables and annual variables, respectively. The studies that suggest the corresponding firm characteristic variables covered in this research are included in parentheses.

No.	Acronym	Definition of the characteristic	No.	Acronym	Definition of the characteristic
1	beta	Estimated market beta from weekly returns and market returns for 3 years ending month t−1 with at least 52 weeks of returns [40].	20	chcsho	Annual percentage change in shares outstanding [58]
2	betasq	Market beta squared [42]	21	currat	Current assets/current liabilities [59]
3	chmom	Difference between the cumulative returns from months t−6 to t−1 and months t−12 to t−7 [43]	22	convind	An indicator equal to 1 if the company has convertible debt obligations [58]
4	high52	The ratio of the current adjusted share price to the highest adjusted share price for the past 52 weeks [44]	23	depr	Depreciation divided by property, plant, and equipment (PP&E) [60]
5	idiovol	The standard deviation of residuals of weekly returns on weekly market returns for three years before the month-end [45]	24	dy	Total dividends divided by market capitalization at the fiscal year-end [61]
6	ill	Average of the daily absolute return/dollar volume [46]	25	egr	Annual percentage change in the book value of equity [63]
7	maxret	Maximum daily return from returns during calendar month t−1 [47]	26	gma	Revenue minus cost of goods sold divided by lagged total assets [1]
8	mom1m	One-month cumulative returns [48]	27	hire	Percentage change in the number of employees [64]
9	mom6m	Five-month cumulative returns ending one month before the month-end [48]	28	lev	Total liabilities divided by fiscal year-end market capitalization [65]
10	mom12m	11-month cumulative returns ending one month before the month-end [49]	29	lgr	Annual percentage change in total liabilities [63]
11	mom36m	Cumulative returns from months t−36 to t−13 [48]	30	pchcurrat	Percentage change in the current ratio [59]
12	mvel1	Natural log of market capitalization at the end of month t−1 [50]	31	pchdepr	Percentage change in depreciation [61]
13	retvol	The standard deviation of the daily returns from month t−1 [51]	32	pchgm_pchsale	Percentage change in gross margin minus percentage change in sales [66]
14	ts	Total skewness of the returns during month t−1 [52]	33	quick	(Current assets−inventory)/current liabilities [59]
15	absacc	The absolute value of accruals [53]	34	pchquick	Percentage change in the quick ratio [59]
16	acc	Annual income before extraordinary items minus operating cash flows divided by average total assets [54]	35	rd_mve	R&D expense divided by fiscal year-end market capitalization [67]
17	agr	Annual percent change in total assets [55]	36	rd_sale	R&D expense divided by sales [67]
18	cash	Cash and cash equivalents divided by average total assets [56]	37	sgr	Annual percentage change in sales [68]
19	cfp	Operating cash flows divided by fiscal year-end market capitalization [57]	38	sp	Annual revenue (sale) divided by fiscal year-end market capitalization [69]

The second category (15–38) includes annual variables: absolute accruals (absacc) [53], accruals (acc) [54], asset growth (agr) [55], cash holdings (cash) [56], cash flow to price ratio (cfp) [57], change in shares outstanding (chcsho) [58], convertible debt indicator (convind) [59], current ratio (currat) [60], depreciation divided by property, plant, and equipment (PP&E) (depr) [61], dividend to price ratio (dy) [62], growth in common shareholder equity (egr) [63], gross profitability (gma) [1], employee growth rate (hire) [64], leverage (lev) [65], growth in long-term debt (lgr) [63], change in current ratio (pchcurrat) [59], change in depreciation (pchdepr) [61], change in gross margin minus change in sales (pchgm_pchsale) [66], change in quick ratio (pchquick) [59], quick ratio (quick) [59], research and development (R&D) expenditure to market capitalization (rd_mve) [67], R&D to sales (rd_sale) [67], sales growth (sgr) [68], and sales to price ratio (SP) [69].

To avoid a forward-looking bias, following Gu *et al*. [10], we match realized returns at month *t* with the most recent monthly variables at the end of month *t−1* and the most recent annual variables as of *t−6*. To eliminate the influence of outliers and facilitate model learning, we normalize all characteristics into the interval *(−1*, *1)* for each month *t* as in Eq (1). Specifically, we calculate the monthly rank based on the cross-section of each firm characteristic variable. Subsequently, we divide this rank by the total number of shares, multiply by two, and subtract one. In this case, we standardize each variable with the maximum value of 1 and the minimum value of −1 (see the normalizing equation below).


$$Z_{i,t}\;=\left[2\;*\frac{Rank\left(Firm\;characteristics\;variable_{i,t}\right)}{N}\right]\;-\;1.$$ (1)

Specifically, we calculate the monthly rank based on the cross-section of each firm characteristic variable. Subsequently, we divide this rank by the total number of shares, multiply by two, and subtract one. In this case, we standardize each variable with the maximum value of 1 and the minimum value of −1 (see the normalizing equation below). The reason for this standardization is that outliers can excessively influence the machine learning model [70].

### 3.2. Conditional autoencoder

The CA structure is based on the arbitrage pricing theory. The static linear factor model used in many studies can be described as in Eq (2) [7, 71].

$$r_{t}=\;\beta \;*\;f_{t}\;+\;e_{t},$$ (2)

where *r*_*t*_ represents the return vector that exceeds the risk-free interest rate, *f*_*t*_ is the vector representing the factor returns of *K × 1*, and *e*_*t*_ is a vector indicating idiosyncratic errors of *N × 1*. Further, *β* is a vector indicating a factor loading of *N × K*, where *N* is the number of items and *K* is the number of factors. This is the same form as the general (standard) factor model used in empirical finance. For example, the Fama and French [30, 72] three-factor (FF3) model uses observable factors in the financial market, such as market return, SMB, and HML. By contrast, Bai and Ng [73] and Stock and Watson [74] examine the latent factor through dimensionality reduction in the return covariance matrix using methods such as PCA.

The PCA model can be used to infer latent factors from returns and obtain dynamically changing coefficients of latent factors. However, the PCA method is an unsupervised learning technique and does not reflect external information, leading to constant betas over time and states. To solve this problem, Kelly *et al*. [9] extend PCA and propose an IPCA model that infers latent factors and dynamically changing betas through external variables, as in Eq (3). Applying this model, the beta varies dynamically by firm characteristics and market environment.


$$r_{i,\;t}=\;\beta_{i,t}\;*\;f_{t}\;+\;e_{t},\;where\;\beta_{i,t}=z_{i,t-1}\;*\;\Gamma .$$ (3)

We use the autoencoder, a generalized version of PCA, to guide dimension reduction. Autoencoder used in this study is one of the deep neural network models and has been introduced in many studies related to dimensionality reduction [75–77]. The main principle of the autoencoder is taken from its name. "Automatic" means that the method is unsupervised learning, and "encoder" means learning different representations of the data. In particular, the autoencoder learns the encoded representation by minimizing the loss between the original data and the decoded data. Therefore, an autoencoder is a neural network that encodes input data into a low-dimensional representation and then decodes it again to train it to map the output data itself. The lower- dimensional representation allows the autoencoder to capture the greatest features of the data. Due to these characteristics, autoencoders can be considered nonlinear generalizations of PCA [78].

**Fig 1:**
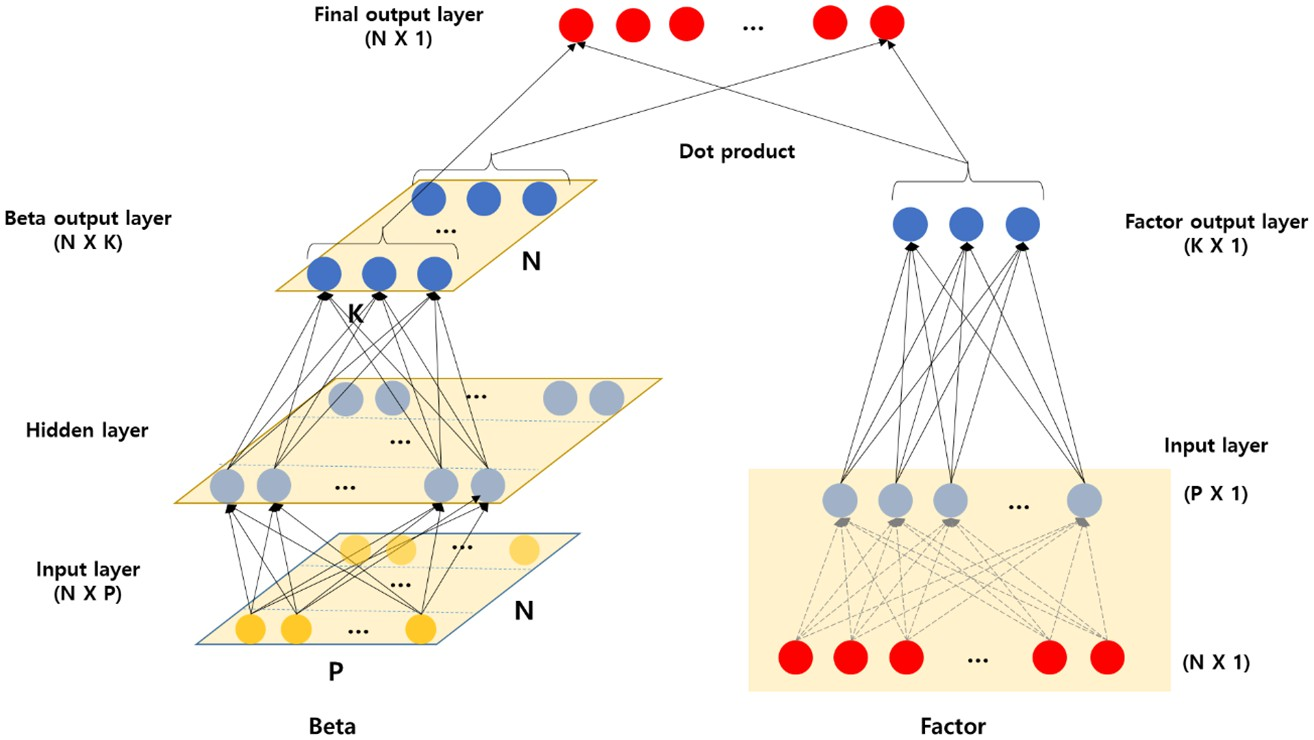
Conditional autoencoder model architecture. This figure describes the conditional autoencoder, which dynamically captures factor loadings and factors. The neural network on the left-hand side generates factor loadings by propagating firm characteristics. The input layer (N × P) comprises assets and firm characteristics in which N is the number of firms and P is the number of firm characteristics. The neural network on the right-hand side generates factors, and its input layer can either be characteristic-managed portfolios or individual asset returns.

Particularly, we apply the CA to infer the factors (*f*_*t*_) and factor loadings (*β*_*i*,*t*_). As illustrated in Eq (3), the CA model allows dynamic factor loading, *β*_*i*,*t*_, in the latent factor through the autoencoder, *f*_*t*_. Further, the model has a nonlinear beta that changes with the firm characteristic variable. *z*_*i*,*t*−1_ represents a company characteristic variable, and matrix *Γ* defines the mapping between many characteristics and a small number of latent factors. The mapping is described in detail in the figure. Fig 1 describes the CA architecture. The left-hand side depicts how the *β* is deduced. In model learning, we use stock returns and firm characteristic variables for *N* stocks over time *T*. As the firm characteristic variables for each stock pass through the hidden layers, they become compressed to *K* dimensions. Note that Fig 1 only illustrates the learning structure at a specific time *t*.

The right-hand side demonstrates the process by which the latent factors are channeled through individual stock returns. The return data of *N* stocks are then compressed into K dimensions on the latent factors. To accomplish this, following the method of Gu *et al*. [10], *N* number of individual stocks constitute *P* number of long-short portfolios based on the values of each *P* firm characteristic variable. This indicates that the data dimension is reduced from *N* to *P* by constructing long-short portfolios (*P*) using firm characteristic variables. As the number of network nodes in the autoencoder model reduces significantly (because of using *P* rather than *N* variables), model learning is facilitated. Additionally, when learning from portfolio returns, the autoencoder mitigates noises generated by individual stock returns. The result, calculated on the left-hand and right-hand sides, is finally dot-produced as *(N × K) × (K × 1)* to create *N × 1* returns.

The CA model has certain advantages compared to the standard autoencoder model illustrated in S1 Fig in S1 Appendix. As the standard autoencoder model has output and input layers identical to the number of stocks, it cannot reflect external market information in beta that can change over time. However, our model depicted in Fig 1 can reflect the external information on the left-hand side of the beta part, and consequently, we can estimate the dynamic betas.

### 3.3. Model learning

We divide the sample data into three disjoint periods, “train,” “validation,” and “test.” Considering the characteristics of time series data, instead of shuffling, we separate the data while maintaining the time order. Specifically, the “train” subsample comprises data for estimating the model according to a specific set of tuning hyperparameter values. The “validation” subsample is used to tune the hyperparameters of the model. Finally, we apply the “test” subsample, which has never been used for “train” or “validation,” to evaluate the method’s OOS performance.

Considering 30 years of the sample period, from January 1991 to December 2020, we set the first 13 years of data as the “train” subsample (1991–2003), the next 2 years as the “validation” subsample (2004–2005), and the remaining 15 years as the “test” subsample (2006–2020). Next, in the machine learning process, we increase the train data by one year over time, and continuously increase and retrain the first train data. Each time we refit the model once a year, we increase the “train” subsample by one year. We maintain the sample size of the “validation” subsample by rolling it forward to include the most recent data.

**Fig 2:**
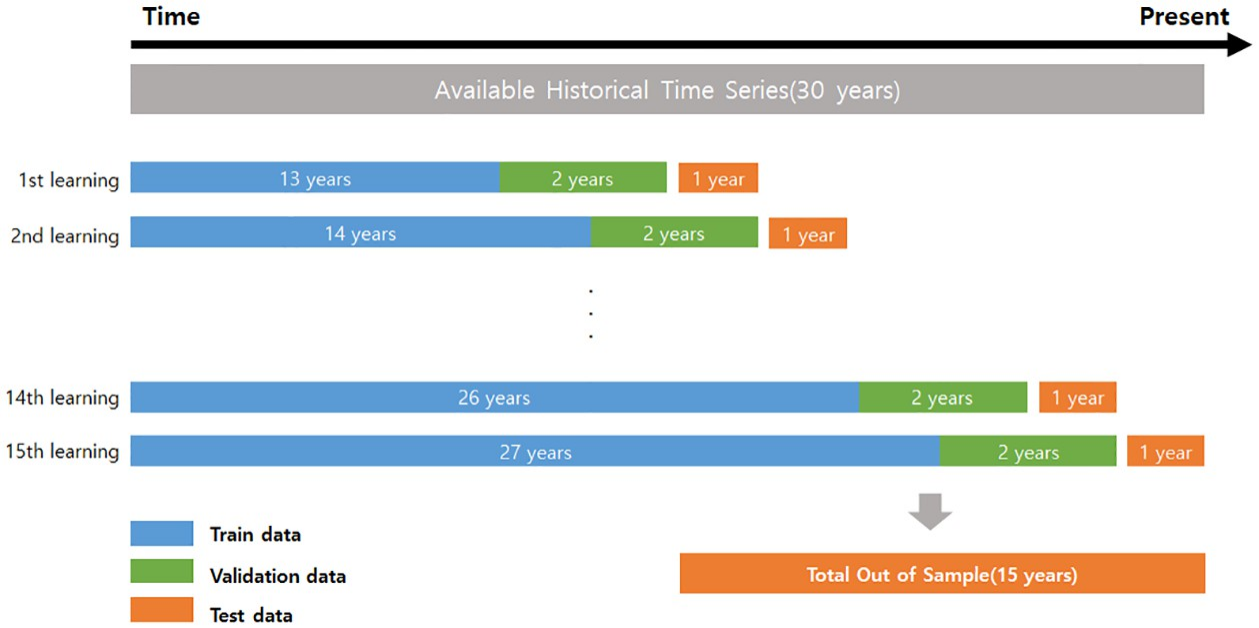
Sliding window model. This figure describes how the model is trained, validated, and tested. First, the model is trained with 13 years of data followed by 2 years of validation data and 1 year of test data. Second, the model is reinitialized and trained with 14 years of data followed by 2 years of validation data and 1 year of test data. This process is iterated up to 15 years of test data.

Fig 2 presents the rolling window of machine learning. In this process, the amount of data used for learning increases over time, which can reflect the changing market conditions in the learning model. For example, our first window with the “test” subsample of 2006 is constructed using the “train” subsample from 1991 to 2003 and the “validation” subsample from 2004 to 2005. Further, the next “test” subsample of 2007 is constructed using the “train” subsample from 1991 to 2004 and the “validation” subsample from 2005 to 2006. Thus, while learning 15 times, 15 years of OOS data are created.

Table 2 displays the hyperparameters used in our CA model learning process. To prevent overfitting, we apply various regularizations during the network training. First, we apply the L1 regularization to the objective function of the neural network model, as in Eq (4).

$$Minimize\frac{1}{NT}\sum_{t=1}^{T}\sum_{i=1}^{N}\;{\left(r_{i,t}-\beta_{i,t-1}f_{t}\right)}^{2}+\phi \;\left(\theta \right),$$ (4)

where *ϕ*(*θ*) refers to the L1 (norm) penalty term, which sets the Laplace distribution to a prior distribution and makes the weights sparse. *r*_*i*_ refers to the actual return of stock *i*, and beta*factor (*β*_*i*,*t*-1_*f*_*t*_) indicates the return estimated by the CA model using *N* stocks over time *T*. The CA model uses Adam optima [79] through the above objective function. Adam optima utilizes low-dimensional moments as one of the stochastic optimization methods and is known to work adequately under non-stationary, noisy, and sparse gradients.

**Table 2:** Model hyperparameters. This table reports the hyperparameters. The hyperparameters are shared between the conditional autoencoder (CA) models except for the number of latent factors (K) and hidden layers. The CA model is specified by setting K and hidden layers.

Hyperparameters	Set values
Layer Initialization Method	Glorot Uniform [81]
L1 Penalty	1e-4
Mini Batch Size	32
Learning Rate	1e-3
Optimizer	Adam
Adam—beta1	0.9
Adam—beta2	0.999
Adam—epsilon	1e-7
Batch Norm—momentum	0.99
Batch Norm—epsilon	1e-3
Early Stopping–patience (Number of epochs with no improvement after which training will be stopped)	500
K	1, 2, 3, 4, 5, 6
Hidden layer	[], [32], [32, 16], [32, 16, 8]
Number of models for ensemble	5

Second, we apply a batch norm that can reduce the effect of layer initialization and solve the covariate shift problem, enabling smoother learning [80, 81]. It reduces the training time and improves the generalization of the model by preventing overfitting.

Third, we apply early stopping—an algorithm that terminates learning when the error of validation data increases after a certain period while training a model with the “train” subsample—using validation data to prevent excessive overfitting.

Finally, we employ the ensemble method, which creates and trains five same-structure models with different random seeds. Given the nature of the neural network, the performance varies depending on the random seed. The ensemble method mitigates the problem and makes the results robust. We obtain the final output of the model by averaging the outputs of the five models.

### 3.4. Model comparison

We compare the performance of the latent factor models as follows. First, we vary the CA model according to the specifications of hidden layers. The CA0 model has no hidden layer. The CA1 model adds a hidden layer of 32 neurons, while the CA2 model adds a hidden layer of 16 neurons to CA1. Further, the CA3 model adds a hidden layer of eight neurons to CA2.

We analyze the FF asset pricing models that are based on the observable factors to enable comparison with the above-mentioned CA models. The explanatory power of the traditional asset and that of the AI-based CA model are compared. The reason to compare these explanatory powers is that the number of factors in the CA model or in the traditional asset pricing model can be naturally controlled.

Table 3 represents the composition of the observable factors in the FF models according to the number K, which we set from one to six to match the number of latent factors created by compressing the dimensions in the CA model. The table illustrates that K = 3 compares to the Mkt-Rf (excess return on the market), SMB (small minus big), and HML (high minus low) factors as the FF3 model. The K = 4 model compares to the Carhart (FF4) model [82] that adds UMD (up minus down) factor to the FF3 model. K = 5 compares to the FF five-factor (FF5) model that adds RMW (robust minus weak) and CMA (conservative minus aggressive) factors to the FF3 factors [2]. Additionally, we add UMD factors to the FF5 factors in six-factor model, which helps compare CA models to the asset pricing models using latent factors or observable factors in correspondence.

**Table 3:** Observable factors according to the number of latent factors.

Number of latent factor (K)	Observable factor
1	Mkt-Rf
2	Mkt-Rf, SMB
3	Mkt-Rf, SMB, HML
4	Mkt-Rf, SMB, HML, UMD
5	Mkt-Rf, SMB, HML, CMA, RMW
6	Mkt-Rf, SMB, HML, CMA, RMW, UMD

This table describes the observable Fama–French (FF) factors according to the number of latent factors (K). Mkt-Rf: the excess return on the market; SMB: small minus big; HML: high minus low; UMD: up minus down; CMA: conservative minus aggressive; RMW: robust minus weak

## 4. Empirical analysis

### 4.1. Asset pricing performance

In this section, we compare the performances of the CA and traditional asset pricing models. We evaluate the OOS performance using the data from January 2006 to December 2020. To verify the explanatory power of the model, we examine both individual stock returns and test portfolios created through firm characteristic variables. For the test portfolio, we construct a portfolio return through a “bottom-up” approach, following Gu *et al*. [24] and Feng *et al*. [83], as in Eq (5).


$$R_{p}=\sum_{i=1}^{N}\;w_{i}\;*\;r_{i,t}.$$ (5)

The portfolio return (*R*_*p*_) is measured by the weighted sum of *r*_*i*,*t*_ of *N* individual stocks. We construct a set of 5 × 5 portfolios by crossing two firm characteristics. Each firm characteristic produces five groups of firms. In the bivariate comparisons, we always include firm size. As our analysis covers 38 firm characteristic variables, we generate a total of 950 portfolios as test assets. We use the total R^2^ to evaluate the performance, as in Eq (6).


$$R_{total}^{2}=1-\frac{\sum_{\left(i,t\right)\in OOS}\;{\left(r_{i,t}-{\hat{\beta }}_{i,t-1}^{\prime }{\hat{f}}_{t}\right)}^{2}}{\sum_{\left(i,t\right)\in OOS\;}r_{i,t}^{2}}.$$ (6)

The total R^2^ ($R_{total}^{2}$) indicates the extent to which the latent factors derived by the model explain the stock return, *r*_*i*_ refers to the actual return, and beta*factor (${\hat{\beta }}_{i,t-1}^{\prime }{\hat{f}}_{t}$) denotes the return estimated by the CA model. In the portfolios, *r*_*i*_ reflects the portfolio return; we can calculate ${\hat{\beta }}_{i,t-1}^{\prime }{\hat{f}}_{t}$ similarly.

Table 4 presents the OOS total R^2^ (%) for individual stocks using the observable factor models (FF) and the CA models (CA0 to CA3). In all cases, the number of factors varies from K = 1 to K = 6. Additionally, the total R^2^ from our CA model generally dominates that from the FF models, which implies that the CA model has more explanatory power. Further, it illustrates that the explanatory power of the CA models increases as the number of factors, K, increases. Interestingly, the total R^2^ of the CA model becomes larger as the number of factors increases, while that of the FF models is not significantly affected by the number of factors. The results imply that, in the case of the FF models, added factors are mitigated by other existing factors. Conversely, in the case of the CA model, the model performance improves linearly with the inclusion of additional factors, suggesting that the model effectively extracts the latent factor information.

**Table 4:** Out-of-sample total R^2^ for individual stocks. This table reports the out-of-sample total R^2^ (%) for individual stocks using observable Fama–French (FF) factor models and conditional autoencoder (CA) models (CA0 through CA3). It presents the results of applying latent factors from 1 to 6 to each model.

Model	1	2	3	4	5	6
FF	6.840	9.504	8.035	6.281	4.579	2.727
CA0	12.104	12.883	13.565	14.197	14.507	14.666
CA1	11.497	12.559	13.248	14.128	14.604	14.818
CA2	11.819	12.880	13.460	14.172	14.578	14.819
CA3	11.808	12.843	13.462	13.998	14.289	14.518

We examine the consistency of these results not only in individual stocks but also at the portfolio level in Table 5. The 5 × 5 portfolio on firm characteristic variables comprises two portfolio types—value-weight (VW) and equal-weight (EW) portfolios. The total R^2^ trend in Table 5 is similar to that of individual stocks in Table 4.

**Table 5:** Out-of-sample total R^2^ for test-asset portfolio. This table reports the OOS total R^2^ (%) for test-asset portfolios using observable Fama–French (FF) factor models and conditional autoencoder (CA) models (CA0 through CA3). It presents the result of applying latent factors (K) from 1 to 6 to each model. Panels A and B illustrate the results from equal-weight and value-weight portfolios, respectively.

Panel A: Equal-weight						
Model	1	2	3	4	5	6
FF	49.927	73.821	74.692	75.094	74.941	75.308
CA0	75.102	78.013	81.412	82.884	83.309	83.232
CA1	73.214	76.680	79.529	82.219	82.979	83.190
CA2	74.302	77.565	80.154	81.701	82.823	83.178
CA3	73.793	77.542	79.804	81.638	82.451	82.828
Panel B: Value-weight						
Model	1	2	3	4	5	6
FF	48.019	69.169	69.351	70.180	68.934	69.694
CA0	67.282	68.962	71.573	72.656	73.316	73.316
CA1	64.885	67.427	69.137	71.858	73.130	72.859
CA2	64.691	67.830	69.795	71.501	73.147	73.293
CA3	63.461	66.875	69.413	71.600	73.340	73.765

First, in the case of EW, the table demonstrates that the explanatory power of the CA model is superior to that of the FF models. Second, the explanatory power of the CA model tends to increase as K increases, regardless of EW and VW portfolio types. In the case of the FF models, VW portfolios display mostly high explanatory power when K exceeds three. Further, the explanatory power increases significantly if K increases from one to two when the FF model adds the SMB factor representing the size effect. This is natural because the VW portfolio represents market-cap-weighted portfolios. Nevertheless, when K increases further, the increase in the explanatory power is relatively low.

**Fig 3:**
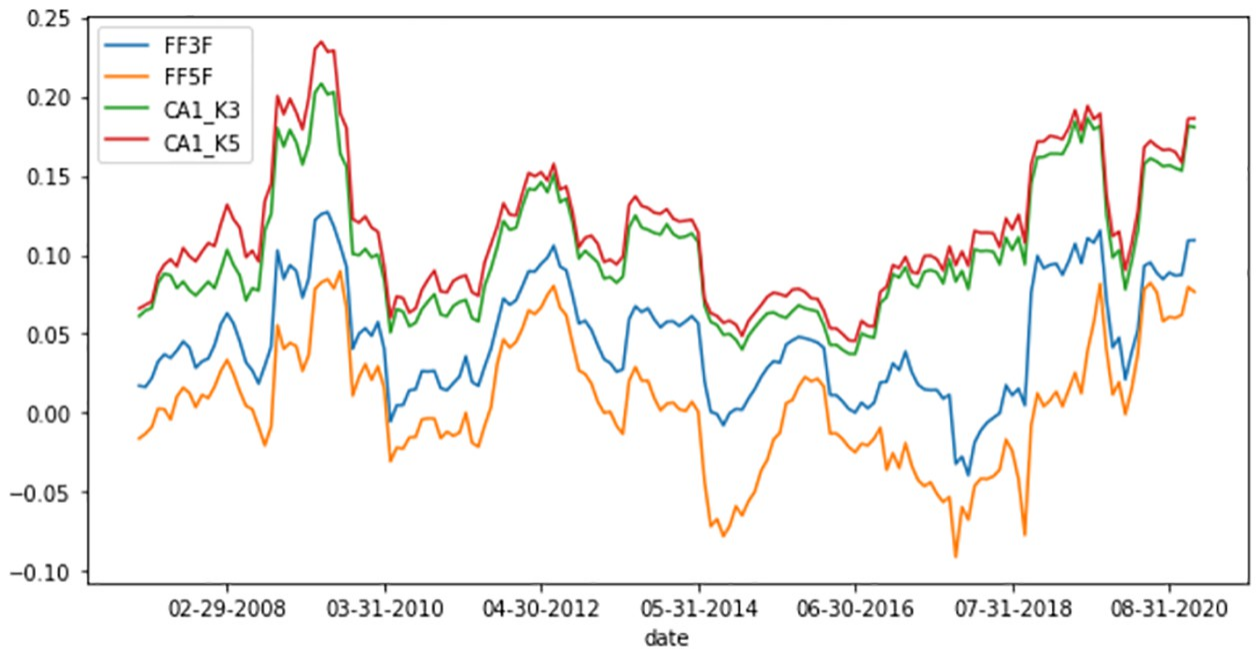
12 months rolling total R^2^. This figure represents the 12-month rolling R^2^ for the FF3F, FF5F, CA1_K3, and CA1_K5 models. Both CA1_K3 and CA1_K5 have higher total R^2^ than FF3F or FF5F over the entire test period. FF: Fama–French; CA: conditional autoencoder.

Furthermore, we examine how the model explanatory power changes at each time point. Fig 3 compares the explanatory power of the CA models with that of the FF3 and FF5 models. For comparison, we consider the CA model with K = 3 and K = 5. In this analysis, R^2^ is the 12-month moving average value using monthly data. In Fig 3, the explanatory power of each model moves differently depending on the time point; however, the CA model is always superior to the FF models. These results confirm that the explanatory power of the CA models is superior regardless of timing or macro situations.

### 4.2. Subsample analysis

Here, we examine the explanatory power of various sub-samples. In this study, we test whether the CA model can provide generalized explanatory power regardless of subsample composition. To this end, in this study, five subsamples of industry classification, market classification, firm size, penny stock, and market inefficient stock classification are examined. To simplify, we set K = 5, which implies that the CA and FF5 models have the same number of factors for comparison.

We tested the explanatory power of the models on subsamples in 4 perspectives. First, we compared the difference of the explanatory powers according to the KOSPI market and the KOSDAQ market because the Korean stock market is largely divided into The KOSPI market and the KOSDAQ market. Second, we examined the difference of the explanatory powers regarding the presence of the penny stocks. Third, we studied whether the investors irrationality affects the explanatory power or not. Finally, we divided the samples randomly, and tested whether the explanatory power of the CA model is robust. The market anomaly can be more pronounced for KOSDAQ-listed stocks. The traditional asset pricing model is known to have low explanatory power for KOSDAQ-listed stocks [84], which is akin to the findings for other international stock markets. This means that the existing asset price model lacks explanatory power in the KOSDAQ market. On the other hand, we test whether the CA model exhibits superior explanatory power in both the KOSPI and KOSDAQ markets.

We examine the difference in the explanatory power between the KOSPI and KOSDAQ markets. Table 6 reports the total R^2^ for individual stocks using the OOS model. The results from Table 6 confirm that the explanatory power for KOSDAQ-listed stocks is relatively low compared to KOSPI-listed stocks in both the FF and CA models.

**Table 6:** Out-of-sample total R^2^ for individual stocks by market.

Model	KOSPI	KOSDAQ	t-value
FF	7.869	2.885	3.510 (0.001)***
CA0	17.231	13.486	3.025 (0.002)***
CA1	17.086	13.688	2.365 (0.019)**
CA2	17.086	13.654	2.732 (0.006)***
CA3	16.792	13.373	2.394 (0.017)**

This table reports the out-of-sample total R^2^ (%) by market segmentation for individual stocks using observable Fama–French (FF) factor models and conditional autoencoder (CA) models (CA0 through CA3). The t-value represents the test value of the monthly total R^2^ difference between the Korea Composite Stock Price Index (KOSPI) and Korea Securities Dealers Automated Quotations (KOSDAQ).

*, **, and *** denote the rejection of the null hypothesis of the absence of causality at the 10%, 5%, and 1% levels, respectively.

In the case of the FF models, the total R^2^ of KOSDAQ-listed stocks (2.885) are 63% lower compared to those of KOSPI-listed stocks (7.869). However, in the case of the CA models, the difference is less dramatic—only a 20% decrease from KOSPI-listed stocks (17%) to KOSDAQ-listed stocks (14%). Overall, while the explanatory power of the CA models decreases in the KOSDAQ market, the CA model still dominates the FF models and exhibits stable performances. The results of this study are consistent with the existing Han *et al*. (2020) studies, as traditional asset price models show low explanatory power in the KOSDAQ market. When analyzing the KOSDAQ market through the existing asset price model, the explanatory power itself is low, so it can be a factor that has many limitations in using the model. However, the CA model has superior explanatory power compared to the existing asset price model, and it implies that the difference in explanatory power between the KOSPI market and the KOSDAQ market is not large. This means that the CA model can provide excellent explanatory power in both markets with different market characteristics. Also, similar results were found in subsamples according to the industry or the company market value. The CA model has excellent explanatory power in detail industries subsamples. In addition, the explanatory power was examined by subsamples according to the company market value and this result is also good regardless of the market value. The result tables describe in detail the explanatory power according to the subsamples, located S1-S3 Tables in S1 Appendix.

Penny stocks tend to have high returns, systematic risk, and unsystematic risk. Therefore, the traditional asset pricing models have low explanatory power over penny stocks. We define penny stocks as those with a closing price of 5,000 won or less in the Korean market, following Kim and Kang [85].

Table 7 illustrates the results with the entire sample, the sample excluding penny stocks, and penny stocks. The FF models indicate that the explanatory power increases when penny stocks are excluded from the sample. However, the CA models suggest no statistically significant differences in terms of penny stocks.

**Table 7:** Out-of-sample total R^2^ for individual stocks with or without penny stocks.

Model	Entire sample	Excluding penny stocks	t-value	Penny stocks	t-value
FF	4.579	6.471	−3.110 (0.002)**	2.876	4.365 (0.000)***
CA0	14.507	13.579	0.278 (0.781)	15.494	−0.021 (0.982)
CA1	14.604	13.541	0.693 (0.489)	15.735	−0.578 (0.563)
CA2	14.578	13.458	0.758 (0.449)	15.770	−0.674 (0.501)
CA3	14.289	13.167	0.592 (0.554)	15.483	−0.592 (0.554)

This table reports the out-of-sample total R^2^ (%) for individual stocks using observable Fama–French (FF) factor models and conditional autoencoder (CA) models (CA0 through CA3). Along with the entire sample, we illustrate the results with and without penny stocks. The t-value represents the test value of the monthly total R^2^ difference from that of the entire sample.

*, **, and *** denote the rejection of the null hypothesis of the absence of causality at the 10%, 5%, and 1% levels, respectively.

As in the results of this study, it can be seen that the explanatory power of penny stock is very insufficient in the traditional asset price model. However, the CA models suggest no statistically significant differences in terms of penny stocks. This means that in the CA model, there is no difference between the explanatory power of penny stock and the explanatory power of other samples. This indicates that, unlike the FF models, the CA model shows excellent explanatory power even in penny stocks. That is, we found that the CA model can provide adequate explanatory power for stock returns with or without penny stocks.

The transaction cost and the irrationality of investors significantly reduce the explanatory power of the asset pricing models for the Korean markets [86]. Therefore, we examine the impact of transaction cost and investors’ irrationality on total R^2^. As a proxy for transaction cost, we use Roll’s spread [87] as in Eq (7), where *cov* represents the auto-covariance of stock returns, using the daily return from t−12 to t−1 months. We convert all positive auto-covariances to negative numbers when calculating *roll spread*, following Roll [87] and Lesmond [88].


$$roll\;spread_{i}=\sqrt{-cov_{i}}.$$ (7)

Another estimate of transaction cost is based on the limited dependent variable model, following Lesmond *et al*. [88]. It includes spread effects, price impact effects, and market depth effects and can be expressed as Eq (8).

$$\begin{array}{c} R\left(i,t\right)=R^{*}\left(i,t\right)-\alpha_{1}\left(i\right),\;if\;R^{*}\left(i,t\right)<\alpha_{1}\left(i\right)\\ R\left(i,t\right)=0,\;if\;\alpha_{1}\left(i\right)\leq R^{*}\left(i,t\right)\leq \alpha_{2}\left(i\right)\\ R\left(i,t\right)=R^{*}\left(i,t\right)-\alpha_{2}\left(i\right),\;if\;R^{*}\left(i,t\right)>\alpha_{2}\left(i\right), \end{array}$$ (8)

where *α*_1_(*i*) < 0 is the sell-side transaction cost of stock *i*, *α*_2_(*i*) > 0 is the buy-side transaction cost, and *R*(*i*, *t*) is the observed actual rate of return. Further, R*(*i*, *t*) is the rate of return in the market without unobserved friction according to the market model regression. Specifically, the observed return is generated by the behaviors of investors considering transaction costs. Additionally, the model assumes that the investor will act (buy, sell) when the expected profit (loss) exceeds the transaction cost. Assuming R*(*i*, *t*) follows a normal distribution, the log-likelihood of the above expression is given by Eq (9).

$$\begin{array}{l} ln\;L\;=\sum_{R_{1}}ln\frac{1}{{\left(2\pi \sigma {\left(i\right)}^{2}\right)}^{\frac{1}{2}}}\\ \;\;\;\;\;\;\;\;\;\;\;\;\;\;\;\;\;\;\;\;\;\;\;-\sum_{1}\frac{1}{2\sigma {\left(i\right)}^{2}}{\left(R\left(i,t\right)+\alpha_{1}\left(i\right)-b\left(i\right)R_{M}\left(t\right)\right)}^{2}\\ \;\;\;\;\;\;\;\;\;\;\;\;\;\;\;\;\;\;\;\;\;\;\;+\sum_{R_{2}}\;ln\frac{1}{{\left(2\pi \sigma {\left(i\right)}^{2}\right)}^{\frac{1}{2}}}\\ \;\;\;\;\;\;\;\;\;\;\;\;\;\;\;\;\;\;\;\;\;\;\;-\sum_{R_{1}}\frac{1}{2\sigma {\left(i\right)}^{2}}{\left(R\left(i,t\right)+\alpha_{2}\left(i\right)-b\left(i\right)R_{M}\left(t\right)\right)}^{2}\\ \;\;\;\;\;\;\;\;\;\;\;\;\;\;\;\;\;\;\;\;\;\;\;+\sum_{R_{0}}\;ln\;ln\;\;\left(\Phi_{2}\left(i\right)-\Phi_{1}\left(i\right)\right)\; \end{array}$$ (9)

where *R*_0_, *R*_1_, and *R*_2_ correspond to the case where the observed rates of return are zero, negative, and positive, respectively. *σ*^2^ is the variance estimated using the observed actual returns, and *Φ* denotes the cumulative distribution function of the standard normal distribution. Estimates of *α*_1_ and *α*_2_ can be obtained by maximizing the above log-likelihood function. *α*_2_(*i*) − *α*_1_(*i*) are estimates of the round-trip transaction cost of competitive and marginal investors. Following Lesmond *et al*. [89], we use *α*_2_(*i*) − *α*_1_(*i*) as the transaction cost.

Finally, we calculate retail composition as a proxy for investors’ irrationality [90, 91]. Individual investors tend to exhibit more behavioral bias in their trading than other investors largely because of overconfidence and disposition effects [90–93]. We calculate retail composition as the trading volume of individual investors relative to the total trading volume, as expressed in Eq (10).


$$Retail\;Composition_{i,t}\;=\frac{Individual\;Trading\;Volume_{i,t}}{Total\;Trading\;Volume_{i,t}}.$$ (10)

Subsequently, we divide our entire sample into two groups using each proxy—high and low—based on the upper and lower 30% in the cross-section. Through the method proposed by Racicot [94], it was shown that the traditional FF lacks the explanatory power of the flow factor, and the explanatory power of the illiquidity can be significant through GMM estimation [95, 96]. For the robustness of the analysis, a liquidity factor [97] is added to the traditional asset price model. We compare the model in which the liquidity factor (LIQ) is added to the asset price model in Table 3 where K is 4 and the model where K is 5. The liquidity factor is a constructed variable. LIQ factor is the average of the stocks *γ*_*i*,*t*_ from regression Eq (11).

$${\text{r}}_{i,d+1,t}-{\text{r}}_{m,d+1,t}=\theta_{i,t}+\varphi_{i,t}{\text{r}}_{i,d,t}+\gamma_{i,t}\text{sign}\left({\text{r}}_{i,d,t}-{\text{r}}_{m,d,t}\right)\nu_{i,d,t}+\varepsilon_{i,d,t}.$$ (11)

where r_*i*,*d*,*t*_ is the return of stock *i* on day *d* in month *t* and v_*i*,*d*,*t*_ is the dollar trading volume of stock *i* on day *d* in month *t*. ε_*i*,*d*,*t*_ is the residual of stock *i* on day *d* in month *t*.

Table 8 demonstrates that the explanatory power of stocks with large investor irrationality and trading restrictions is statistically significantly lower than that of the others. Regarding the magnitude of the reduced explanatory power, FF5 displays negative explanatory power in the subsamples with large transaction costs and investor irrationality. This implies that most of the explanatory power of FF5 disappears. Adding the liquidity factor increases the explanatory power of highly liquid stocks, but still lowers the explanatory power of illiquid stocks. As argued by Racicot and Rentz [95], the LIQ factor in the FF model supports that the explanatory power is not large. On the other hand, the CA model exhibits an explanatory power of 8–10%. This indicates that the explanatory power of the traditional asset price model is still inferior to that of CA even when the LIQ factor is considered. In S4 Table in S1 Appendix, various cases are examined, such as adding the LIQ factor to FF5F according to the method of Racicot and Rentz [95]. In all cases, the main results are consistent.

**Table 8:** Out-of-sample total R^2^ for individual stocks by transaction cost and investors’ irrationality.

Model	High	Low	t-value
Panel A: Roll’s spread			
FF5F(K = 5)	<0	1.500	-3.048 (0.002)***
FF4F(K = 4)+LIQ	<0	1.9143	-3.289 (0.001)***
CA0	9.767	12.843	-4.8545 (0.000)***
CA1	10.046	13.035	-4.581 (0.000)***
CA2	10.055	13.128	-4.783 (0.000)***
CA3	9.778	12.701	-4.508 (0.000)***
Panel B: Lesmond transaction cost			
FF5F(K = 5)	<0	0.093	-1.2145 (0.226)
FF4F(K = 4)+LIQ	<0	1.6271	-1.5865 (0.114)
CA0	9.266	13.012	-5.771 (0.000)***
CA1	9.582	13.114	-5.725 (0.000)***
CA2	9.541	13.183	-6.019 (0.000)***
CA3	9.485	13.059	-5.716 (0.000)***
Panel C: Retail composition			
FF5F(K = 5)	<0	3.792	-2.9288 (0.003)***
FF4F(K = 4)+LIQ	0.008	4.547	-3.248 (0.001)***
CA0	8.609	12.335	-3.159 (0.002)***
CA1	8.927	12.091	-2.961 (0.007)***
CA2	8.889	12.122	-2.770 (0.008)***
CA3	8.619	11.613	-2.521 (0.012)**

This table reports the out-of-sample (OOS) total R^2^ (%) for individual stocks using observable Fama–French (FF) factor models and conditional autoencoder (CA) models (CA0 through CA3). Panels A, B, and C represent the OOS total R^2^ of the samples classified according to Roll’s spread, Lesmond transaction cost, and retail composition size, respectively. The t-value represents the test value of the monthly total R^2^ difference.

*, **, and *** denote the rejection of the null hypothesis of the absence of causality at the 10%, 5%, and 1% levels, respectively.

In conclusion, despite the factors that lower the explanatory power of the asset pricing models for the Korean market, the explanatory power of the CA models remains considerably robust compared to that of the FF models. This means that the time-varying model is an effective systematic risk measure [96], and the CA model fits this purpose well and shows excellent explanatory power even for stocks with low illiquidity.

This result confirms that the performance of the asset price model deteriorates as the investor irrationality and transaction limiting factors increase in the Korean market, which is consistent with Chae and Yang’s [86] findings. On the other hand, the CA model shows excellent explanatory power in all samples. This shows that the CA model overcomes the limitations that the existing asset price model failed in the Korean market. This shows that the CA model can be a model for explaining the stock returns in the Korean market.

To check the robustness, following Gu *et al*. [10], we retrain and refit the CA model using subsamples of stocks composed of odd and even tickers, respectively. Each odd and even ticker is composed of 1,612 subsamples, and these tickers do not mutually overlap in any sample. To check the robustness, we test the odd and even samples in the OOS after training the model using only the odd sample. Conversely, we test the odd and even samples in the OOS after training the model using only even samples. This verification method is advantageous in that it enables checking the robustness of the model despite omission or arbitrary deformation of the sample. Table 9 illustrates the results.

**Table 9:** Robustness test. This table reports the out-of-sample (OOS) total R^2^ (%) for the subsamples of stocks that have odd and even permanent numbers, based on parameters estimated separately. The rows present the subsample {Even, Odd} for which we estimate the parameters, whereas the columns represent the subsample {Even, Odd} for which we evaluate the OOS performance. All estimates are based on the five-factor CA1 model. CA: conditional autoencoder.

Total R^2^ (%)		Test	
		Even	Odd
Training	Even	11.24	11.62
	Odd	11.94	10.87

The model trained with even samples exhibits 11.24% of the total R^2^ for even samples and 11.62% for odd samples in the OOS. Further, the model trained with odd samples displays 11.94% of the total R^2^ for even samples, and 10.87% for odd samples in OOS. Overall, the total R^2^ is similar in both methods. This indicates that the explanatory power of the CA model is stable even when some samples are omitted in the model training process.

### 4.3. Portfolio alpha test

Now, we directly examine whether the CA model can explain the market anomalies using portfolio alpha tests. Specifically, following Gu *et al*. [10], we test whether the average of the residuals for each long-short portfolio created based on 38 firm characteristics is statistically different from zero.

To construct an estimate of the pricing error from the OOS data, we compute the mean difference between the actual return and the model estimation. The difference between the two values can be interpreted as the alpha (pricing error, *α*) of each portfolio, as in Eq (12).


$$\alpha =E\left(r_{i,t}\right)-E\;\left(\beta_{i,t-1}f_{t}\right).$$ (12)

We analyze the existence of alpha using the CA, FF3, and FF5 models. For comparison, we employ an identical number of factors used in each comparative model. For example, we compare the FF3 model with the CA model with K = 3, and the FF5 model with the CA model with K = 5.

After composing the deciles portfolio according to the magnitude of the firm characteristic variables, we measure the alpha of the 10–1 long-short portfolio. We construct both VW and EW portfolios and judge the significance of alpha based on the t-values of 1.96 and 2.58 according to the 95% and 99% significance levels, respectively.

**Fig 4:**
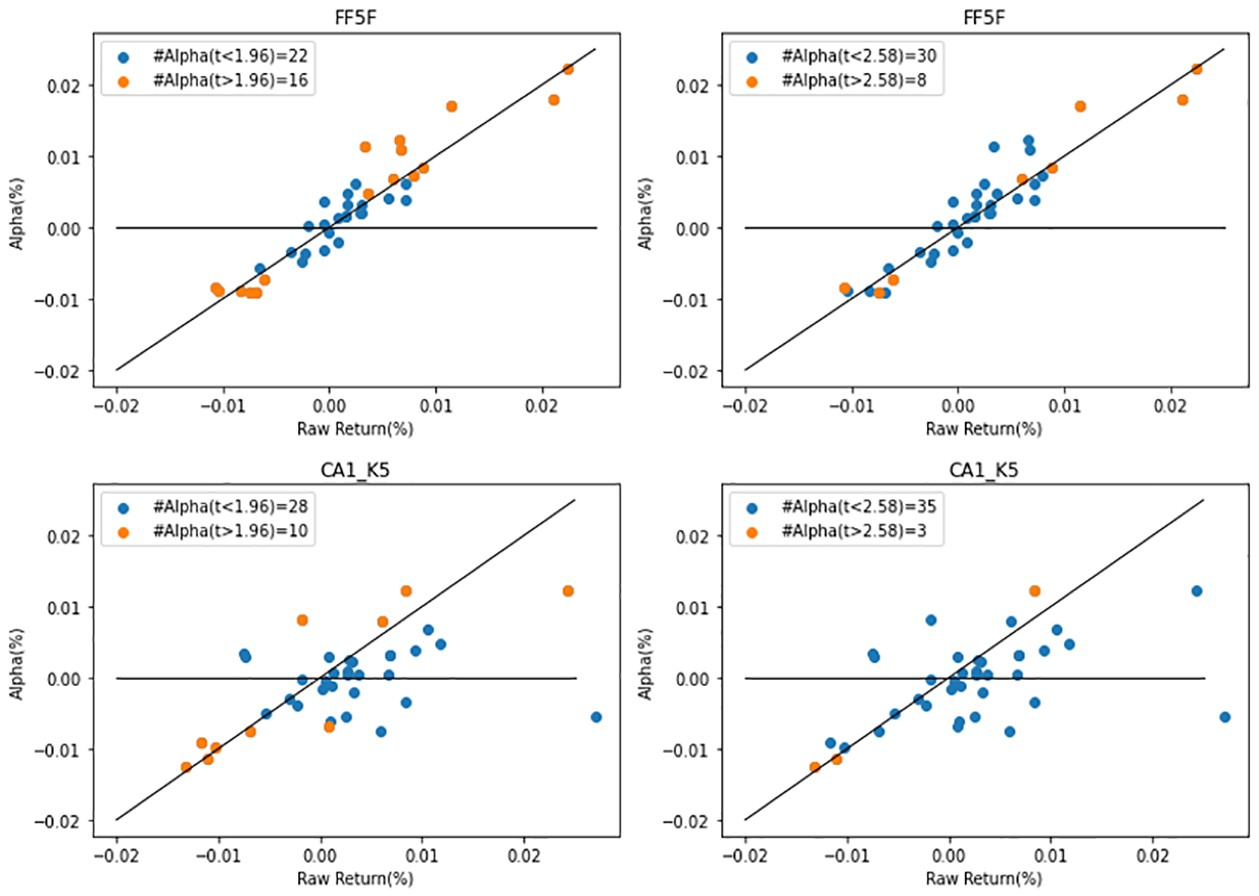
Out-of-sample alpha. The figure reports the out-of-sample pricing errors (alphas) for 38 characteristic-managed portfolios, relative to the Fama–French (FF) factor model and conditional autoencoder (CA) model (CA1). Alphas with t-statistics more than 1.96 and 2.58 are depicted as orange dots, while insignificant alphas are represented by blue dots.

Fig 4 plots the alpha and average return of the VW portfolio. The plots on the left show the number of alphas with a t-statistic exceeding 1.96, and those on the right represent the number of alphas with a t-statistic exceeding 2.58. For the FF5 model, 16 out of 38 portfolios have alphas above 1.96 and 8 out of 38 portfolios have alphas above 2.58. However, the CA model with K = 5 reduces the number of alphas to 10 or 3 depending on the cutoff standard of 1.96 and 2.58.

Table 10 represents the full results. Compared to the traditional asset pricing model, when measuring alpha through the CA model, it can be seen that the number is reduced. Also, in the case where the cutoff is 2.58, where the alpha measurement is strict, the number of CAs tends to decrease as K increases. This is a trend that appears in both EW and VW. Comprehensively, the CA models have superior power in explaining portfolio alphas or market anomalies compared to the traditional asset pricing models.

**Table 10:** Number of alphas. This table reports the number of alphas using test portfolios and the observable Fama–French (FF) factor model in Panel A and the conditional autoencoder (CA) model in Panel B. Alpha is the mean difference between the actual return and the model estimation. The significance of alpha is judged based on the t-values of 1.96 and 2.58 according to the 95% and 99% significance levels, respectively.

Model	Equal-weight		Value-weight	
	cutoff = 1.96	cutoff = 2.58	cutoff = 1.96	cutoff = 2.58
Panel A: Traditional (Observable) asset pricing model				
K = 1	24	17	12	4
K = 2	25	17	13	5
K = 3	25	20	16	6
K = 4	24	20	13	8
K = 5	25	22	16	8
K = 6	24	20	14	6
Mean	24.5	19.3	14	6.2
Median	24.5	20	13.5	6
Panel B: Conditional Autoencoder model				
K = 1	26	20	13	9
K = 2	20	12	10	6
K = 3	17	11	8	5
K = 4	17	13	7	4
K = 5	15	12	10	3
K = 6	10	8	10	3
Mean	17.5	12.6	9.6	5
Median	17	12	10	4.5

Intuitively, the presence of alphas explained by the CA models implies that omitted (unobserved) risk factors beyond the FF factors possibly generate excess returns. This indicates that the CA model effectively reflects factors that explain stock returns in addition to the factors dealt with in the traditional asset price model. Therefore, it can be said that the conditional autoencoder structure used in this study has a great advantage in deriving the latent factor from market data. These pricing errors can be interpreted as the average gain of a long-short portfolio that has zero exposure for any systematic factors. That means, the anomaly rate of return due to company characteristic variables can be explained by risk factors as claimed by Gu *et al*. [10], and it demonstrates that the CA model can explain these returns well.

### 4.4. APT strategy

To examine whether the CA model is a better fit for the Korean stock market compared to the traditional asset pricing model, we compare the long-short profits based on the expected return calculated by the traditional asset price model and our latent factor model, the CA model, respectively.

**Fig 5:**
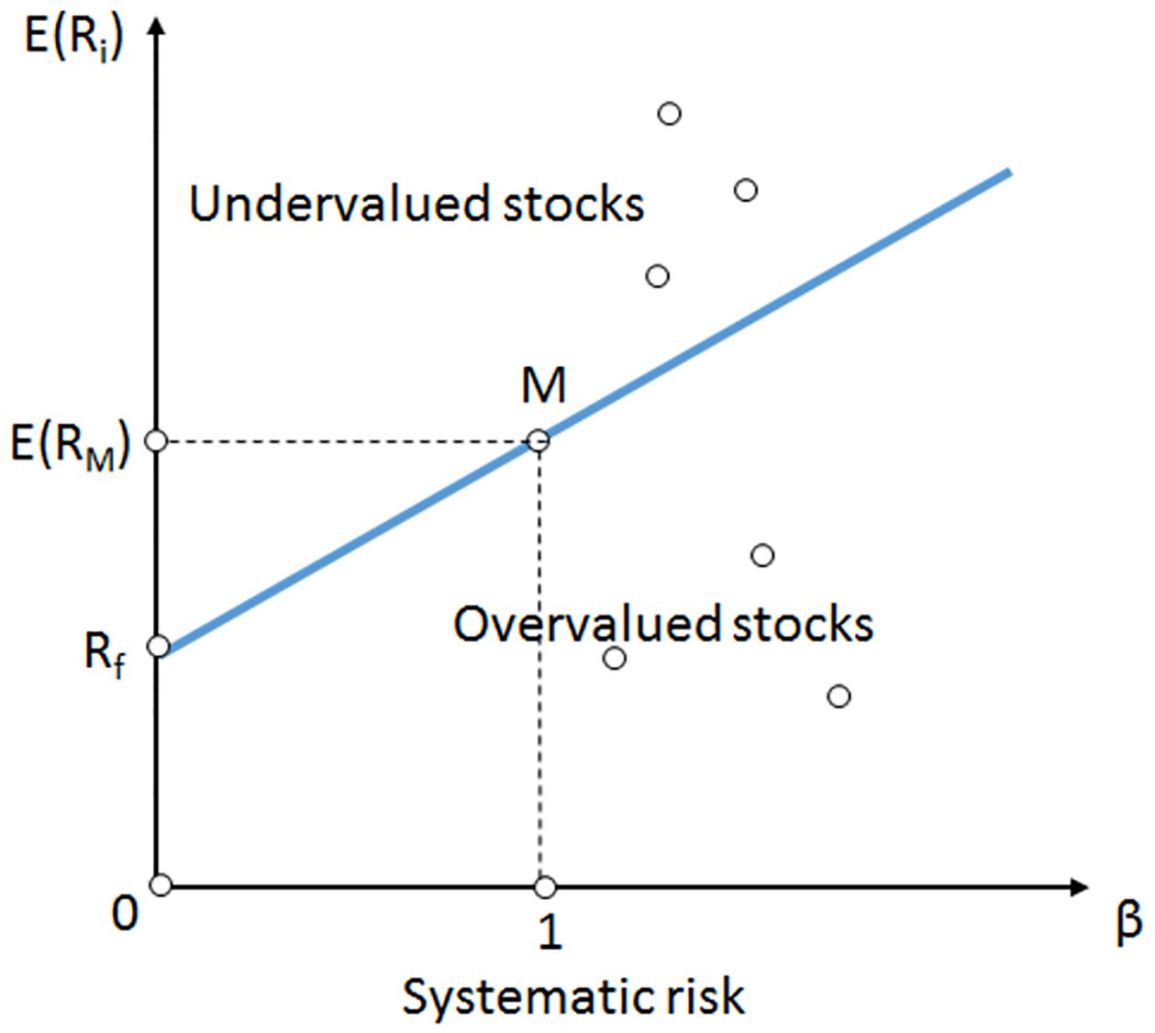
CAPM model.

Fig 5 shows undervalued or overvalued stocks for the CAPM model. The expected return of stocks lying on the SML line satisfies the CAPM model, which has the intrinsic value. The undervalued (overvalued) stock is plotted above (below) the SML line, whose actual Ri is larger than E[R].


$$\begin{array}{l} Undervalued\;stock:\;E\left(r_{i,t}\right)-r_{i,t}>0\\ overvalued\;stock:\;E\left(r_{i,t}\right)-r_{i,t}<0 \end{array}$$ (13)

Similarly, the multi-factor asset pricing model is expressed as Eq (13). In this chapter, we compare the expected return calculated by the traditional asset price model and our latent factor model, the CA model, respectively.


$$E\left(r_{i,t}\right)=r_{f}+\beta_{1}\;*\;factor_{1}+\beta_{2}\;*\;factor_{2}+\cdots +u_{t}$$ (14)

$$\begin{array}{l} Undervalued\;stock:\;E\left(r_{i,t}\right)-r_{i,t}>cut\;off\;value\\ overvalued\;stock:\;E\left(r_{i,t}\right)-r_{i,t}<cut\;off\;value \end{array}$$ (15)

The pricing error is calculated as the difference between the expected rate of return and the actual rate of return. To eliminate the trivial error value, we set the reference level of the average pricing error at 1% using the past 1-month window. For example, the undervalued (overvalued) stock is classified when the average value of the difference between the expected return and the actual return for the last month is larger than 1% (-1%).

Then, we construct a long-short portfolio with undervalued stocks and overvalued stocks. Traditional asset pricing models to calculate the expected return includes CAPM, FF3F, FF4F and FF5F (in Table 11), and the CA models includes CA1, CA3, CA4 and CA5 (in Table 12).

**Table 11:** APT strategy in the traditional asset pricing model. This table presents the performance of each portfolio, from January 2006 to December 2020. The High, M, Low, and LS denotes the rate of return for undervalued stocks, overvalued stocks, stocks lying on SML, and Long-short portfolio, respectively. The monthly values are displayed and parentheses are t-statistics. Classify undervalued stocks as high, overvalued stocks as low, and other stocks as M.

	CAGR	STD	Sharpe	MDD	WIn
Panel A: CAPM					
High	0.0074 (1.5519)	0.0638	0.1157	-0.603947	0.5611
M	0.0064 (1.6197)	0.053	0.1207	-0.5402	0.5611
Low	0.0049 (1.0636)	0.0623	0.0793	-0.687797	0.5444
LS	0.0024 (1.1983)	0.0273	0.0893	-0.186536	0.5444
Panel B: FF3F					
High	0.0075 (1.5902)	0.0634	0.1185	-0.598929	0.5611
M	0.006 (1.5326)	0.0528	0.1142	-0.563535	0.5889
Low	0.0048 (1.0228)	0.0626	0.0762	-0.697518	0.5556
LS	0.0027 (1.3466)	0.0273	0.1004	-0.195603	0.5667
Cahart 4F					
High	0.0075 (1.5992)	0.0631	0.1192	-0.598685	0.5667
M	0.0067 (1.663)*	0.054	0.124	-0.552845	0.5889
Low	0.0047 (0.9982)	0.0627	0.0744	-0.700096	0.5389
LS	0.0029 (1.4065)	0.0272	0.1048	-0.206984	0.5556
FF5F					
High	0.0073 (1.5525)	0.0633	0.1157	-0.600758	0.5722
M	0.0074 (1.8219)*	0.0542	0.1358	-0.523686	0.5722
Low	0.0046 (0.9747)	0.0626	0.0726	-0.698915	0.5556
LS	0.0028 (1.3806)	0.027	0.1029	-0.187524	0.5611

*** p-value < 0.001,

** p-value < 0.01,

* p-value < 0.05

**Table 12:** APT strategy in CA model. This table presents the performance of each portfolio, from January 2006 to December 2020. The Overvalued, Neutral, Undervalued, and LS denotes the rate of return for overvalued stocks, undervalued stocks, stocks lying on SML, and Long-short portfolio, respectively. The monthly values are displayed and parentheses are t-statistics. Classify undervalued stocks as high, overvalued stocks as low, and other stocks as M.

	CAGR	STD	Sharpe	MDD	WIn
CA1					
Undervalued	0.0109 (2.2151)*	0.0655	0.1656	-0.585393	0.5698
Neutal	0.0111 (2.5854)*	0.0574	0.1932	-0.547282	0.6145
Overvalued	0.008 (1.7718)	0.0607	0.1324	-0.610669	0.5698
LS	0.0028 (1.5781)	0.0239	0.118	-0.210341	0.5531
CA3					
Undervalued	0.0119 (2.4261)*	0.0658	0.1813	-0.579546	0.5754
Neutal	0.0119 (2.7746)**	0.0574	0.2074	-0.519233	0.5922
Overvalued	0.0067 (1.4826)	0.0606	0.1108	-0.62031	0.5698
LS	0.0052 (2.9096)**	0.024	0.2175	-0.167702	0.5922
CA4					
Undervalued	0.0124 (2.5236)*	0.0657	0.1886	-0.575966	0.5754
Neutal	0.0114 (2.5975)*	0.0585	0.1941	-0.557836	0.5922
Overvalued	0.0063 (1.3916)	0.0606	0.104	-0.620188	0.5587
LS	0.0061 (3.3581)***	0.0242	0.251	-0.143759	0.6034
CA5					
Undervalued	0.0128 (2.6159)**	0.0657	0.1955	-0.569527	0.5866
Neutal	0.0117 (2.691)**	0.058	0.2011	-0.523682	0.5866
Overvalued	0.0058 (1.2713)	0.0606	0.095	-0.635276	0.5587
LS	0.0071 (3.9757)***	0.0238	0.2972	-0.11571	0.6369

*** p-value < 0.001,

** p-value < 0.01,

* p-value < 0.05

The results from Table 11 shows the results from the strategy performance in the traditional asset pricing model. The Overvalued, Neutral, Undervalued, and LS denotes the rate of return for overvalued stocks, undervalued stocks, stocks lying on SML, and Long-short portfolio, respectively. The results show that the Long-Short strategy profit is not significant for all strategies using the pricing error of each model, including CAPM and FF5F. Even the rate of return from undervalued stocks is not statistically significant. Considering that the definition of High is the stocks whose price would decrease and its expected return would increase until it is plotted exactly on the line, our results indicate that the traditional asset price model is not valid for investment in the Korean market. These results are consistent with Kim and Kim [98] and Kang and Jang [99].

The results from Table 12 show the long-short profit based on undervalued and overvalued stocks in the CA model. The results show that the rate of return from undervalued stocks is statistically significant, unlike the results using the traditional asset pricing model. Also, the results from the Long-Short strategy are statistically significant except for the CA1 model, which has a latent factor of 1. In addition, our results confirm that the latent factor increases, the performance of the LS strategy and the significance level increase. These imply that the overall explanatory power of the model increases as the number of latent factors in the CA model increases.

Since the performance of the strategy reviewed in this study can vary depending on the cut-off value and lookback for pricing error, we examine this. First, the cutoff value for pricing error is set to 3%, 5%, and 10% instead of the existing 1% to examine the trend of changing performance.

Table 13 shows portfolio trends examined by diversifying the cut off value for pricing error. Similar to Table 13, it can be seen that the LS yield increases from CA1 to CA5, and the significance level increases. In particular, it shows that the higher the cut off value, the higher the rate of return of the LS strategy. This means that when a strategy is taken based on stocks with large pricing errors, the effect increases.

**Table 13:** Robust test for cutoff value. This table presents the performance of each portfolio, from January 2006 to December 2020. The Overvalued, Neutral, Undervalued, and LS denotes the rate of return for overvalued stocks, undervalued stocks, stocks lying on SML, and Long-short portfolio, respectively. The monthly values are displayed and parentheses are t-statistics.

	Undervalued	M Neutral	Overvalued	LS
Panel A: cutoff = 0.03				
CA1	0.0109(2.1639)*	0.0116(2.6965)**	0.007(1.5248)	0.0038(1.8577)
CA3	0.0117(2.3285)*	0.0121(2.7696)**	0.0061(1.3047)	0.0056(2.6817)**
CA4	0.0124(2.4604)*	0.0118(2.7346)**	0.0053(1.1409)	0.0071(3.3893)***
CA5	0.013(2.6044)**	0.0115(2.6468)**	0.0047(1.0025)	0.0084(4.1095)***
Panel B: cutoff = 0.05				
CA1	0.0108(2.1122)*	0.0119(2.718)**	0.0058(1.2287)	0.005(2.1936)*
CA3	0.012(2.3549)*	0.0114(2.5973)*	0.0052(1.0881)	0.0069(2.9419)**
CA4	0.0126(2.4521)*	0.0114(2.6043)**	0.0044(0.9262)	0.0082(3.4709)***
CA5	0.0133(2.6087)**	0.0113(2.5524)*	0.0038(0.7953)	0.0096(4.1117)***
Panel C: cutoff = 0.10				
CA1	0.0104(1.9258)	0.0112(2.4982)*	0.0041(0.8206)	0.0063(2.1749)*
CA3	0.0128(2.3661)*	0.0109(2.4238)*	0.0026(0.5109)	0.0103(3.4491)***
CA4	0.0138(2.5267)*	0.0108(2.4064)*	0.002(0.4006)	0.0117(3.8802)***
CA5	0.0143(2.6221)**	0.0108(2.3964)*	0.0015(0.3044)	0.0128(4.2925)***

*** p-value < 0.001,

** p-value < 0.01,

* p-value < 0.05

### 4.5. Importance rankings of firm characteristics

Here, we identify the top 10 most important firm characteristics ex-post. To simplify, we set K = 5 in the CA model. We rank the importance of the characteristics by estimating the reduction in the total R^2^ resulting from setting the values of a given characteristic to zero while holding the remaining estimates fixed. We can estimate the relative importance by standardizing the value of the reduction in the total R^2^ of each variable to [0, 1]. Subsequently, we rank the standardized value to check the importance order of the variables in the CA model. This case has a problem in that the importance of another variable may converge to zero when the extent of change in the R^2^ of a specific variable is large. Fig 5 displays the top 10 most important variables in each CA model (K = 5) based on the average of the entire sample. Overall, market equity (mvel1), total return volatility (retvol), sales to price ratio (SP), and market beta (beta) are commonly selected as important variables for the model.

**Fig 6:**
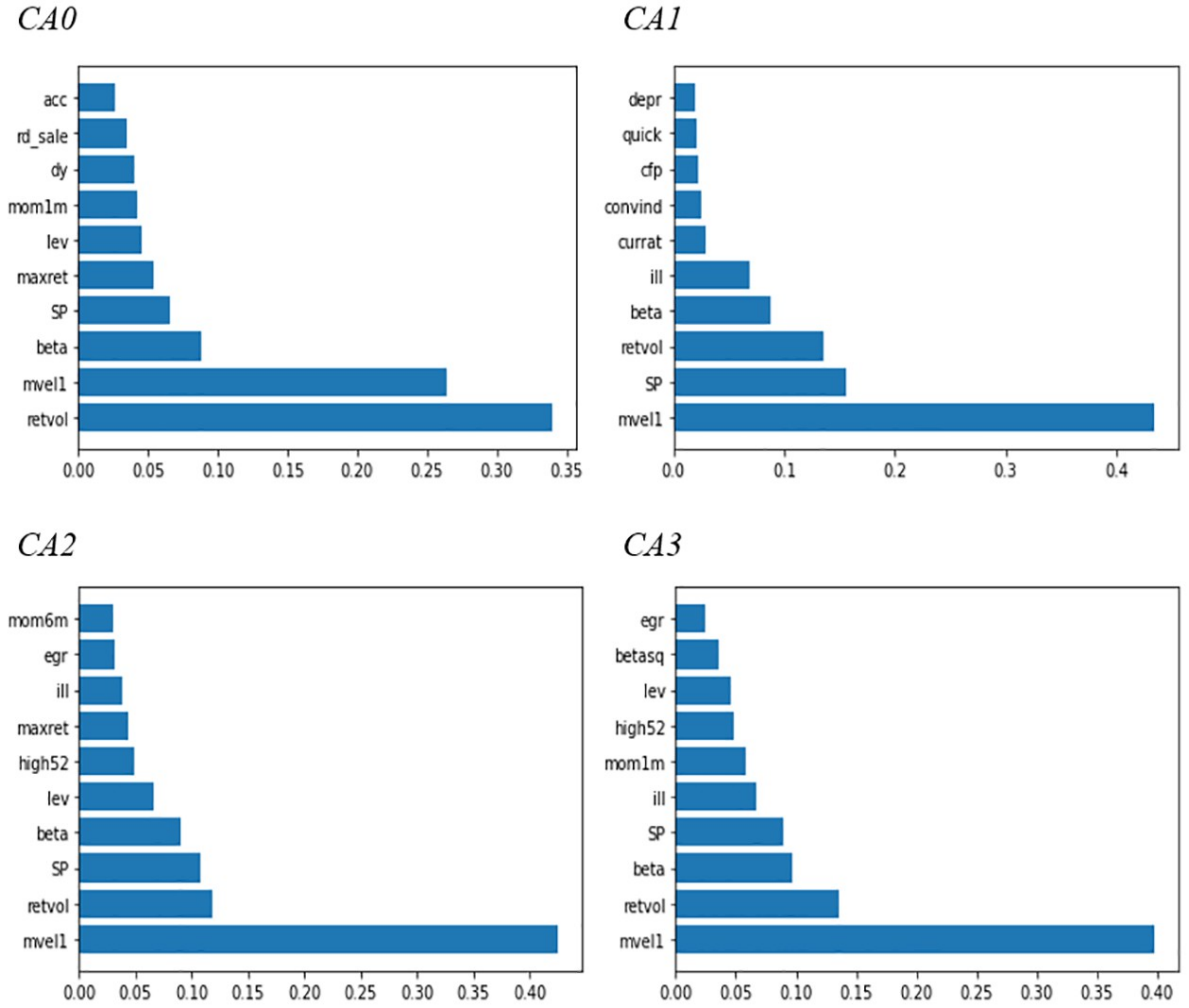
Top 10 characteristic importance. This figure compares variable importance for the top 10 most influential firm characteristics in each model. Each importance within each model is normalized to sum to one. We set the number of latent factors (K) to five for comparison. CA: conditional autoencoder; acc: accruals; rd_sale: R&D to sales; dy: dividend to price ratio; mom1m: 1-month momentum; lev: growth in long-term debt; maxret: maximum daily return; SP: sales to price ratio; beta: market beta; mvel1: market equity; retvol: return volatility; depr: depreciation divided by PP&E; quick: quick ratio; cfp: cash flow to price ratio; convind: convertible debt indicator; currat: current ratio; ill: illiquidity; mom6m: 6-month momentum; egr: growth in common shareholder equity; high52: the ratio of the current price to the 52-week high price; betasq: market beta squared.

Additionally, Fig 6 depicts the importance rankings for all characteristics in each CA model with K = 5. The darker the color, the higher the variable importance. In contrast to Fig 5, the rank values are displayed in the order of importance. In our case, the importance of the top three variables is high. We plot Fig 6 considering that the values of the lower-importance variables are all close to zero when the importance of the variable is expressed as a relative value because the importance of the top three variables is high.

The importance of firm characteristic variables in each model is similar. Particularly, the top five high-importance variables and the bottom five low-importance variables are similar in each model.

When the characteristic importance is calculated through the CA models, different results may be obtained depending on the process of model fitting. We fit several parameters because the autoencoder is a type of neural network, and the model may overfit or underfit depending on hyperparameters. Nevertheless, the characteristic importance in the autoencoder model is useful in assessing the latent factors. For instance, the CA model has important implications for identifying significant firm characteristic variables in asset pricing. By employing these, we can identify the factors that affect asset prices when the market changes rapidly, such as in a financial crisis. Therefore, we now examine the change in the importance of firm characteristics in the subsample through the CA models.

**Fig 7:**
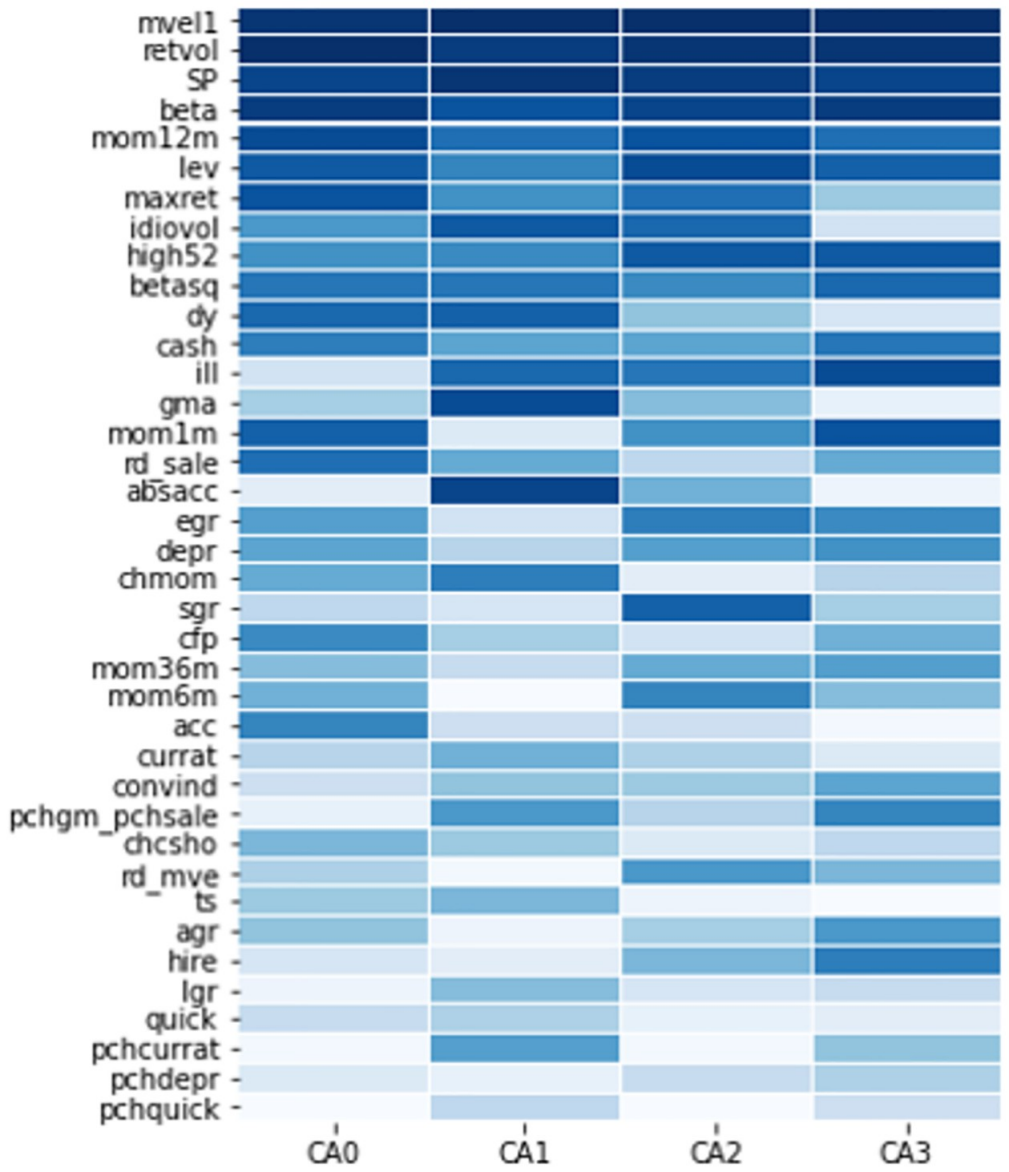
Characteristic importance rank. This figure ranks 38 stock-level characteristics in terms of overall model contributions. The columns correspond to individual models according to the number of hidden layers. The firm characteristic variables are sorted based on the rank sum for the conditional autoencoder model with K = 5. The most important characteristics are at the top and the least influential at the bottom. Additionally, the darker the color, the greater the influence of that variable. mvel1: market equity; retvol: return volatility; SP: sales to price ratio; beta: market beta; mom12m: 12-month momentum; lev: growth in long-term debt; maxret: maximum daily return; idiovol: idiosyncratic return volatility; high52: the ratio of the current price to the 52-week high price; betasq: market beta squared; dy: dividend to price ratio; cash: cash holdings; ill: illiquidity; gma: gross profitability; mom1m: 1-month momentum; rd_sale: R&D to sales; absacc: absolute accruals; egr: growth in common shareholder equity; depr: depreciation divided by PP&E; chmom: change in 6-month momentum; sgr: sales growth; cfp: cash flow to price ratio; mom36m: 36-month momentum; mom6m: 6-month momentum; acc: accruals; currat: current ratio; convind: convertible debt indicator; pchgm_pchsale: change in gross margin minus change in sales; chcsho: change in shares outstanding; rd_mve: expense to market capitalization; ts: total skewness; agr: asset growth; hire: employee growth rate; lgr: growth in long-term debt; quick: quick ratio; pchcurrat: change in current ratio; pchdepr: change in depreciation; pchquick: change in quick ratio; CA: conditional autoencoder.

We set the global financial crisis as the period from July 2007 to June 2009 and the COVID-19 pandemic from January 2020 to December 2020. Fig 7 depicts the importance of the top 10 most influential variables during the financial crisis and the COVID-19 pandemic periods and demonstrates that market equity (mvel1) always has the highest importance. Furthermore, growth in long-term debt (lev) and gross profitability (gma) exhibit high importance during the financial crisis. This finding is in line with the existence of a large proportion of stocks with negative gross profit during the financial crisis (27.9%). However, in the case of the COVID-19 period, illiquidity (ill), the ratio of the current price to the 52-week high price (high52), idiosyncratic return volatility (idiovol), and R&D expense to market capitalization (rd_mve) display high importance. This reflects the growing importance of market friction factors and tech stocks during 2020.

**Fig 8:**
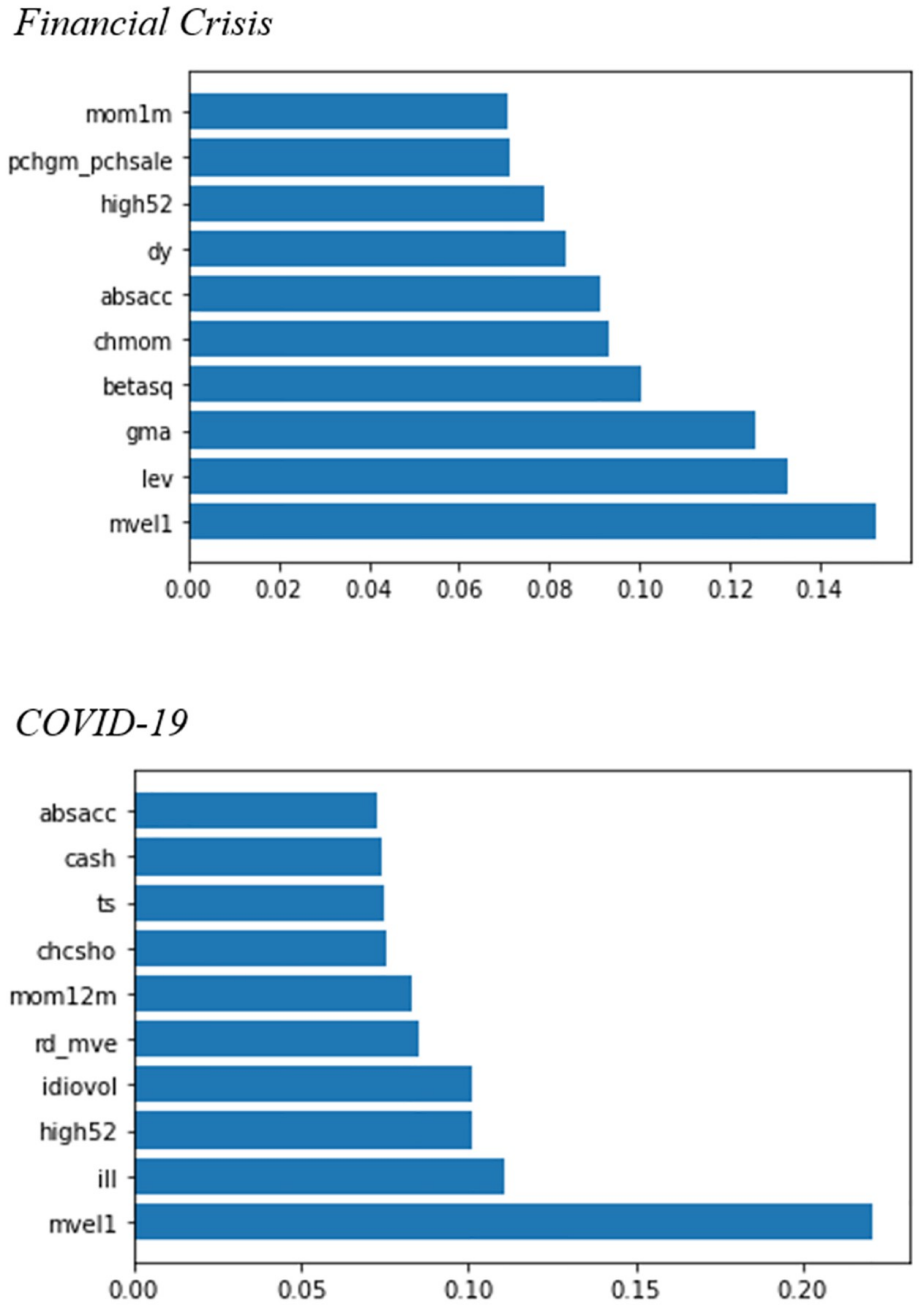
Top 10 most influential firm characteristics. This figure compares variable importance for the top 10 most influential variables in each model. The variable importance within each model is normalized to sum to one. We set the number of the latent factors (K) to five for comparison. mom1m: 1-month momentum; pchgm_pchsale: change in gross margin minus change in sales; high52: the ratio of the current price to the 52-week high price; dy: dividend to price ratio; absacc: absolute accruals; chmom: change in 6-month momentum; betasq: market beta squared; gma: gross profitability; lev: growth in long-term debt; mvel1: market equity; cash: cash holdings; ts: total skewness; chcsho: change in shares outstanding; mom12m: 12-month momentum; rd_mve: expense to market capitalization; idiovol: idiosyncratic return volatility; ill: illiquidity.

The results in Fig 8 show that different variables are considered influential in the CA model for different times. That is, compared to the factors defined in advance in the traditional asset price model, the latent factor by the CA model indicates that the variable market environment can be reflected. This can be said to be the biggest characteristic of the CA model, and it can be inferred that the CA model has superior explanatory power compared to the FF model in this study. It is expected that more diverse analyzes and applications will be possible in the future by deriving important variables of the CA model.

## 5. Conclusion

We applied machine learning-based CA asset pricing models to the Korean stock market. The autoencoder—one of the popular machine learning methods—was employed to extract latent factors, following Gu *et al*. [10]. The autoencoder generalizes PCA by including nonlinearity and is known to effectively extract latent factors and obtain dynamically changing coefficients of latent factors. Thus, the CA model can reflect external market information in financial applications.

We examined the explanatory power of the CA model for the Korean market. Subsequently, we compared the CA model with the traditional asset pricing model. Our results demonstrated that the CA model dominates the traditional models (e.g., the FF models) in terms of OOS R^2^ and stability under various settings including KOSDAQ, small stocks, penny stocks, illiquid stocks and irrational investors’ stocks. This result shows that the CA model can provide generalized explanatory power in markets other than the US market, which is widely used in asset price model studies. Also, as a result of subsample analysis, the existing asset price model has a difference in explanatory power depending on the sample, whereas the CA model shows excellent explanatory power for several subsamples. This indicates that the CA model sufficiently supplements the limitations of the existing asset price model, which lacks explanatory power in a specific subsample. This indirectly shows the structural advantage of deep learning in which the importance of input data dynamically changes through a hidden layer when estimating the latent factor of the CA model.

The CA model can explain several market anomalies that the FF models are unable to clarify. In other words, it shows that the latent factor has a common market risk factor that has not been considered in the existing asset price model. In addition, by using the pricing error of the asset pricing model, strategies based on overvalued stocks or undervalued stocks were compared. The comparison reveals that the performance of the CA model was excellent. This means that the CA model can accurately determine whether a stock is overvalued or undervalued compared to traditional asset pricing models. Thus, the CA model explains the expected returns of stocks well. Lastly, the CA model also revealed the firm characteristics that are important in asset pricing and how their importance varies with macro-financial states. This is the advantage of being able to identify variables that had a significant impact in the entire sample period or a specific period through the CA model. In addition, it shows that the importance of each variable is changed over time, which is a major feature of the CA model. This is a big difference from the model in which the factor is fixed and defined in advance like the FF model. Due to these characteristics, it can be inferred that the CA model shows superior explanatory power compared to the existing asset pricing model.

This study provides several research possibilities. First, trading strategies can be devised using the CA model. Second, more international studies beyond the Korean and U.S. markets are needed. Third, another machine learning-based dimension reduction technique can be compared with the CA model. Fourth, while we focus on an equity market, fixed income and other asset classes can also be analyzed.

## Supporting information

S1 Appendix (DOCX)
