# Correction: Conditional autoencoder asset pricing models for the Korean stock market

**DOI:** 10.1371/journal.pone.0320348

**Published:** 2025-03-19

**Authors:** Eunchong Kim, Taehee Cho, Bonha Koo, Hyoung-Goo Kang

Fig 5 was incorrectly included and should be omitted.

In the APT strategy subsection of the Empirical analysis, there are errors in the second paragraph.

The correct paragraph is: We classify stocks into two categories: upward potential stocks (undervalued stocks) and downward potential stocks (overvalued stocks), based on a comparison of their expected returns with actual returns. Upward potential stocks are those whose expected returns exceed their actual returns, indicating additional profit potential and making them suitable as buy candidates. Conversely, downward potential stocks are those whose expected returns are lower than their actual returns, suggesting a likelihood of decline and making them appropriate as sell candidates.

## References

[1] Kim E, Cho T, Koo B, Kang H-G. Conditional autoencoder asset pricing models for the Korean stock market. PLoS One. 2023;18(7):e0281783. doi: 10.1371/journal.pone.0281783 37523358 PMC10389732

