# Supplementary material for: Conditional autoencoder asset pricing models for the Korean stock market
Source: PLoS One. 2023 Jul 31;18(7):e0281783. doi: 10.1371/journal.pone.0281783 (PMC10389732; doi:10.1371/journal.pone.0281783)
Supplement: S1 Appendix — (DOCX) [file pone.0281783.s001.docx]

Appendix.

S1 Fig. Methodology


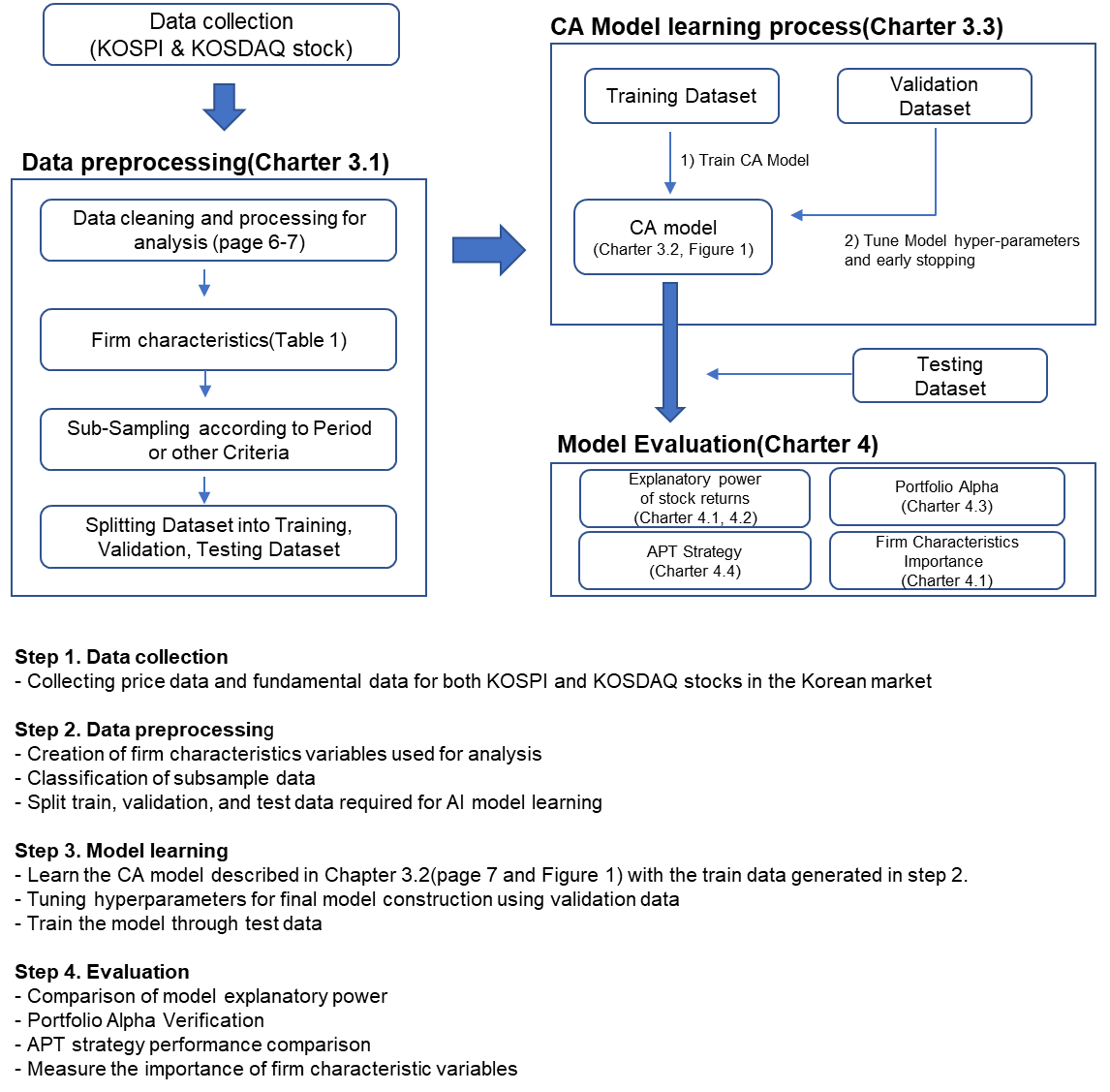


S2 Fig. Autoencoder architecture

This figure represents the conventional autoencoder neural network. Input data are transformed to the encoded data on the latent space through the encoder network. Typically, the dimension of the latent space is smaller than that of the input space. Encoded data are propagated to the decoder network to generate the reconstructed data.


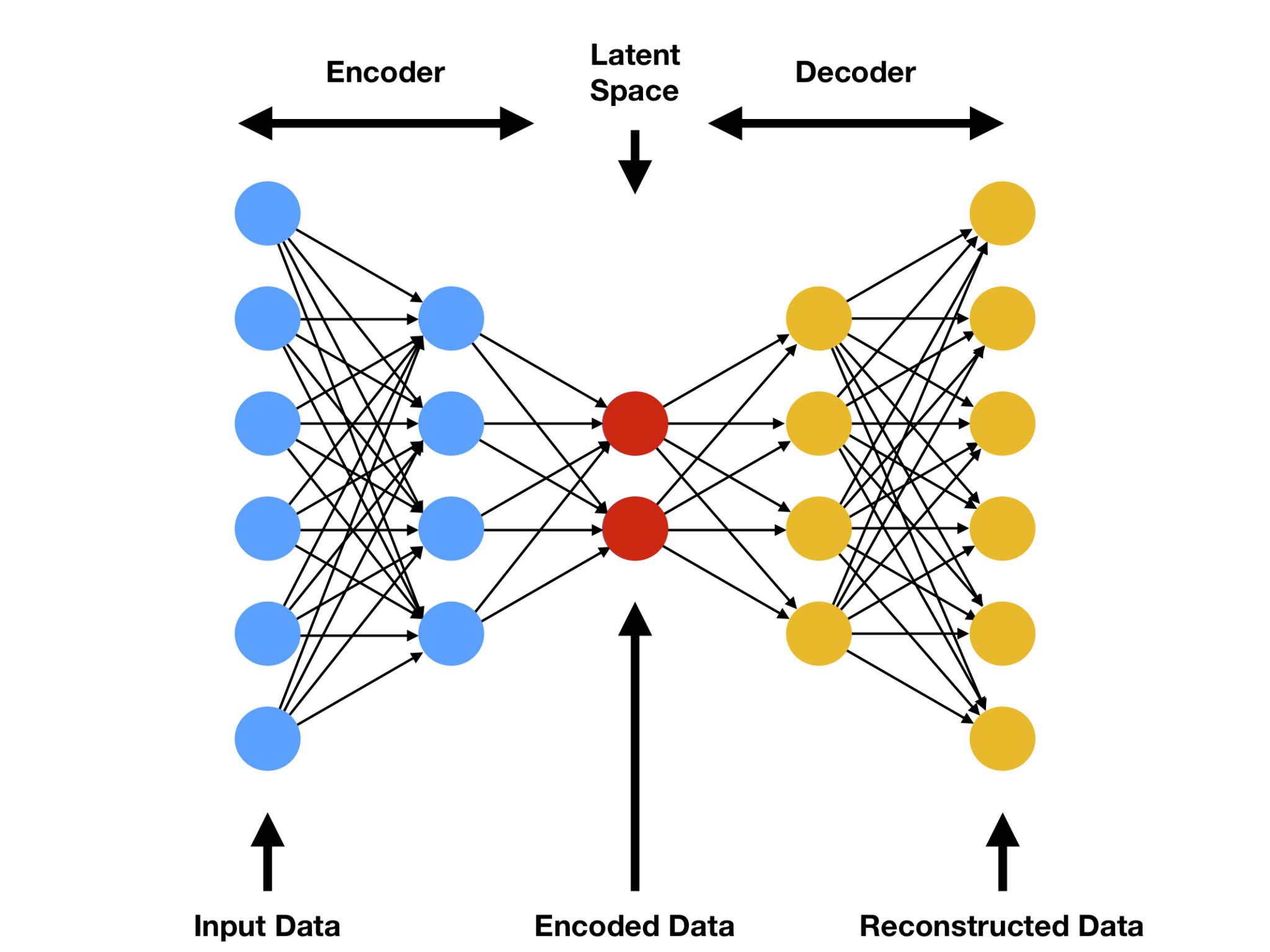


S1 Table. Out-of-sample total R^2^ for individual stocks by sector

This table reports the out-of-sample total R2 (%) for individual stocks using observable Fama–French (FF) factor models and conditional autoencoder (CA) models (CA0 through CA3) by 10 industry sectors.

| Model | FF | CA0 | CA1 | CA2 | CA3 |
| --- | --- | --- | --- | --- | --- |
| IT | 5.374 | 15.004 | 15.104 | 15.021 | 14.767 |
| Materials | 4.129 | 14.312 | 14.457 | 14.403 | 14.098 |
| Health Care | 4.338 | 14.436 | 14.546 | 14.537 | 14.282 |
| Consumer Discretionary | 4.931 | 15.052 | 15.101 | 15.112 | 14.799 |
| Consumer Staple | 4.871 | 14.441 | 14.522 | 14.517 | 14.224 |
| Industrials | 4.119 | 14.002 | 14.130 | 14.095 | 13.781 |
| Communication Services | 4.596 | 14.538 | 14.628 | 14.607 | 14.311 |
| Energy | 4.641 | 14.524 | 14.614 | 14.596 | 14.305 |
| Financials | 4.393 | 14.395 | 14.490 | 14.467 | 14.184 |
| Utilities | 4.564 | 14.507 | 14.606 | 14.574 | 14.290 |

S2 Table. Out-of-sample total R^2^ for individual stocks by firm size

This table reports the out-of-sample total R^2^ (%) according to the firm size classification for individual stocks using observable Fama–French (FF) factor models and conditional autoencoder (CA) models (CA0 through CA3). The t-value represents the test value of the monthly total R^2^ difference between large and small firms. *, **, and *** denote the rejection of the null hypothesis of the absence of causality at the 10%, 5%, and 1% levels, respectively.

| Model | Large | Small | t-value |
| --- | --- | --- | --- |
| FF | 8.781 | 3.469 | 3.925 (0.000)*** |
| CA0 | 15.108 | 14.276 | 0.723 (0.470) |
| CA1 | 15.189 | 14.379 | 0.667 (0.505) |
| CA2 | 15.036 | 14.402 | 0.361 (0.717) |
| CA3 | 14.699 | 14.131 | 0.678 (0.498) |

S3 Table. Out-of-sample total R^2^ for individual stocks with or without penny stocks

This table reports the out-of-sample total R^2^ (%) for individual stocks using observable Fama–French (FF) factor models and conditional autoencoder (CA) models (CA0 through CA3). Along with the entire sample, we illustrate the results with and without penny stocks. The t-value represents the test value of the monthly total R^2^ difference from that of the entire sample. *, **, and *** denote the rejection of the null hypothesis of the absence of causality at the 10%, 5%, and 1% levels, respectively.

| Model | Entire sample | Excluding penny stocks | t-value | Penny stocks | t-value |
| --- | --- | --- | --- | --- | --- |
| FF | 4.579 | 6.471 | −3.110 (0.002)** | 2.876 | 4.365 (0.000)*** |
| CA0 | 14.507 | 13.579 | 0.278 (0.781) | 15.494 | −0.021 (0.982) |
| CA1 | 14.604 | 13.541 | 0.693 (0.489) | 15.735 | −0.578 (0.563) |
| CA2 | 14.578 | 13.458 | 0.758 (0.449) | 15.770 | −0.674 (0.501) |
| CA3 | 14.289 | 13.167 | 0.592 (0.554) | 15.483 | −0.592 (0.554) |

S4 Table. Out-of-sample total R^2^ for individual stocks by transaction cost and investors’ irrationality

This table reports the out-of-sample (OOS) total R^2^ (%) for individual stocks using observable Fama–French (FF) factor models and conditional autoencoder (CA) models (CA0 through CA3). Panels A, B, and C represent the OOS total R^2^ of the samples classified according to Roll's spread, Lesmond transaction cost, and retail composition size, respectively. The t-value represents the test value of the monthly total R^2^ difference. *, **, and *** denote the rejection of the null hypothesis of the absence of causality at the 10%, 5%, and 1% levels, respectively.

| *K* | *Factor Model* | High | Low | t-value |
| --- | --- | --- | --- | --- |
| *Panel A: Roll’s spread* | | | | |
| *4* | *MKT SMB HML UMD* | 0.832 | 3.972 | -3.679(0.000)*** |
| *4* | *MKT SMB HML LIQ* | 0.791 | 4.510 | -4.388(0.000)*** |
| *5* | *MKT SMB HML RWM CMA* | <0 | 1.5000 | -3.048(0.002)*** |
| *5* | *MKT SMB HML UMD LIQ* | <0 | 1.914 | -3.289(0.001)*** |
| *6* | *MKT SMB HML RWM CMA UMD* | <0 | <0 | -2.133(0.034)** |
| *6* | *MKT SMB HML RWM CMA LIQ* | <0 | <0 | -2.759(0.000)*** |
| *Panel B: Lesmond transaction cost* | | | | |
| *4* | *MKT SMB HML UMD* | 1.413 | 4.118 | -2.952(0.003)*** |
| *4* | *MKT SMB HML LIQ* | 1.478 | 4.416 | -2.657(0.001)*** |
| *5* | *MKT SMB HML RWM CMA* | <0 | 0.093 | -1.215(0.226) |
| *5* | *MKT SMB HML UMD LIQ* | <0 | 1.627 | -1.587(0.114) |
| *6* | *MKT SMB HML RWM CMA UMD* | <0 | <0 | -0.249(0.803) |
| *6* | *MKT SMB HML RWM CMA LIQ* | <0 | <0 | 0.052(0.957) |
| *Panel C: Retail composition* | | | | |
| *4* | *MKT SMB HML UMD* | 1.268 | 6.123 | -3.598(0.000)*** |
| *4* | *MKT SMB HML LIQ* | 1.595 | 7.108 | -4.260(0.000)*** |
| *5* | *MKT SMB HML RWM CMA* | <0 | 3.792 | -2.923(0.003)*** |
| *5* | *MKT SMB HML UMD LIQ* | <0 | 4.547 | -3.248(0.001)*** |
| *6* | *MKT SMB HML RWM CMA UMD* | <0 | 0.910 | -1.935(0.054)** |
| *6* | *MKT SMB HML RWM CMA LIQ* | <0 | 1.975 | -2.617(0.001)*** |
